# Effect of Pesticides on Peroxisome Proliferator-Activated Receptors (PPARs) and Their Association with Obesity and Diabetes

**DOI:** 10.1155/2023/1743289

**Published:** 2023-02-24

**Authors:** J. Hernández-Valdez, A. Velázquez-Zepeda, J. C. Sánchez-Meza

**Affiliations:** Facultad de Química, Universidad Autónoma del Estado de México. P. Colon S/N, Residencial Colon and Col Ciprés, Toluca de Lerdo 50120, Mexico

## Abstract

Obesity and diabetes mellitus are considered the most important diseases of the XXI century. Recently, many epidemiological studies have linked exposure to pesticides to the development of obesity and type 2 diabetes mellitus. The role of pesticides and their possible influence on the development of these diseases was investigated by examining the relationship between these compounds and one of the major nuclear receptor families controlling lipid and carbohydrate metabolism: the peroxisome proliferator-activated receptors (PPARs), PPAR*α*, PPAR*β*/*δ*, and PPAR*γ*; this was possible through *in silico*, *in vitro*, and *in vivo* assays. The present review aims to show the effect of pesticides on PPARs and their contribution to the changes in energy metabolism that enable the development of obesity and type 2 diabetes mellitus.

## 1. Introduction

According to the World Health Organization (WHO), obesity affected 13 million people in 2016, and the trend of increasing prevalence is constant, affecting adults and children, regardless of race and social status [1]. Obesity is defined as the loss of balance between the body's energy intake and consumption, leading to the storage of adipose tissue that exceeds its activity and causes hypertrophy and the growth of ectopic adipose tissue [2]. One of the main complications resulting from this metabolic alteration is the development of insulin resistance, leading to type 2 diabetes mellitus. Diabetes mellitus is among the leading causes of death worldwide, ranking ninth in 2019, and was the direct cause of 1.5 million deaths. In large part, these patients were overweight and sedentary. Diabetes mellitus is defined as “a chronic disease that occurs either when the pancreas does not produce enough insulin or when the body cannot effectively use the insulin that is produced” [3]. Both diseases affect a large proportion of the world's population and are interrelated. Obesity is one of the most important risk factors for the development of type 2 diabetes mellitus.

Lately, many epidemiological studies have linked exposure to environmental toxicants such as phthalates, bisphenols, and pesticides to obesity and diabetes [4, 5]. The environmental toxicants capable of promoting lipid accumulation and adipogenesis are known as obesogens; some examples are pesticides such as DDT (dichloro diphenyl trichloroethane), DDE (dichloro diphenyl dichloroethylene), HCH (hexachlorocyclohexane), and chlorpyrifos [6, 7]. On the other hand, most pesticides are endocrine disruptors that alter lipid and carbohydrate metabolism, causing insulin resistance and thus diabetes mellitus [8]. Pesticides such as DDT, DDE, aldicarb, and carbaryl have been linked to the occurrence of diabetes mellitus [9, 10].

Using numerous omics techniques, exposure to pesticides has been linked to the genetic expression of this disease, specifically to one of the key nuclear receptors that control lipid and carbohydrate metabolism: the peroxisome proliferator-activated receptors (PPARs) [6]. PPARs are a family of nuclear receptors of the type II. These receptors bind to a co-repressor protein and when they bind to a ligand, they require a co-activator protein [11], which then forms a complex between the receptor-ligand and retinoid X receptor (RXR), to form a heterodimer, this migrates into the nucleus and binds to the peroxisome proliferator response elements (PPRE), which consist of a sequence of two hexanucleotides (5'-AGGTCA-3') separated by one nucleotide [11, 12]; they enable the transcription of genes that have this sequence in their promoter. Three subtypes have been described so far: PPAR*α*, PPAR*β*/*δ*, and PPAR*γ*. PPAR*α* (also known as NRIC1) was originally identified as an orphan receptor activated by peroxisome proliferation [13]. PPAR*β* or PPAR *δ* (NR1C2) and PPAR*γ* (NR1C3) have been cloned as activator receptors of many proximal proliferators [14, 15]. PPAR*γ* has two alternative promoters that generate two isoforms expressed in different tissues: PPAR*γ*1 in many different tissues and PPAR*γ*2 specifically in adipose tissue, but this expression can also be induced in other organs by a high-fat diet [15, 16].

The aim of the present review is to describe the differences involved in the activation of PPARs by many pesticides, leading to alterations in fat and carbohydrate metabolism, which could contribute to the development of diseases such as obesity and type 2 diabetes mellitus. To this end, three groups of studies have been made, first *in silico* studies that help to predict the binding and interaction between the pesticides and the PPARs; second *in vitro* studies to describe the possible mechanism of action to activate the receptor in specific cell lines; and third *in vivo* studies to evaluate the global response and the simultaneous use of different pathways in complete organisms.

## 2. *In Silico* Predictions: Interaction between Pesticides, Peroxisome Proliferators (PP), and the Peroxisome Proliferator-Activated Receptors (PPARs)

One of the most important points to consider is the description of the docking of the pesticides to the PPAR receptors and their subsequent activation. The interaction of different peroxisomal proliferators (PP) and their PPARs receptors has led to the study of the molecular properties of PP and the active sites of the receptors, this to a better understanding of the binding of both molecules. The *in silico* experiments have been shown to be sufficient to predict these interactions. They show not only the probability of binding between pesticides and the receptor by software such as ToxCast® [17] or AutoDock Vina® [18], but also the molecular interactions between amino acid residues of PPARs involved in binding and stability of ligand-receptor binding, which promotes better activation.  Table 1 summarizes the reports on the prediction of binding and interaction of many pesticides and PPARs, as well as the molecular structures of the pesticides and the software used for prediction.

The use of mathematical and computational tools such as quantitative structure-activity relationship (QSAR) models has enabled the prediction of binding between the pesticide and PPARs, for example, fluazinam, a diarylamine used as a fungicide, with the human PPAR*γ* receptor [17]; fomesafen, an herbicide belonging to the nitrobenzamide group, with the PPAR*α* receptor of mice and rats [19]; these interactions considered their molecular chemical characteristics and their physicochemical and biological properties, mainly the size and flexibility of the molecule, electronic distribution, hydrophobicity, hydrogen bonds, and the presence of many pharmacological features related to biological activity.

The application of predictive docking between ligands and receptors has allowed us to assess the molecular level of interactions between the amino acid residues of the active site of the receptor with moieties or functional groups of the structures of pesticides. In the binding of fomesafen and PPAR*α*, the amino acid residues lysine (Lys) is involved in the electrostatic interactions, and methionine (Met), leucine (Leu), and phenylalanine (Phe) favor *π*–*π* interactions [19]. Other pesticides evaluated by docking include diflubenzuron, a benzoylurea that inhibits chitin synthesis and is an agonist of the human PPAR*γ* receptor interacting through 18 amino acid residues, highlighting cysteine (Cys 285) [20]. Cys 285 was determined by X-ray crystallography to be essential for the binding between organotin, triphenyltin (TPT), and tributyltin (TBT) with PPAR*γ*, which does not favor a covalent ionic interaction between the tin (Sn) of the pesticides and the sulfur (S) in the ionic state of the amino acid [21]. Besides, the antagonistic interaction between rat PPAR*γ* and bromuconazole, a triazole used as a fungicide, must be due to a close interaction between hydrogen bonds formed between the pesticides and the histidine (His 477) of the receptor, which shares the same amino acid with an anchorage that the pharmacological antagonist GW9662 [18]. So it is being shown that the interaction of some amino acids that are constantly involved in pesticide and receptor binding.

As for the structure of the pesticides described earlier, all of them have the same aromatic ring, except for TBT, which has a lower ability to activate PPAR*γ*. However, its ability to ionize allows stability in the ligand–receptor interaction to produce an ion–*π* or *π*–*π* interaction [18–21], as shown in Table 1. As many authors have suggested the use of this predictive technique makes it possible to define amino acid residues and the moieties and/or functional groups of the pesticides that can facilitate receptor activation, activation levels, possibly biological activity, and identification of their behavior as agonist or antagonist.

## 3. *In Vitro* Studies: Binding, Activation, and Mechanism of Action of Pesticides via PPARs Receptors

Cell lines have been the most used to study the binding and biological activation of a ligand to this receptor. They also have the advantage of being accessible in their elaboration and facilitate the understanding of the phenomenon of ligand–receptor integration, so that many pathways can be proposed. The biological effect of the binding of pesticides to the different PPARs, as agonists or antagonists, as well as the biological biomarkers related to the secretion of proteins [22] and/or gene expression [23] transactivated by these nuclear receptors have made it possible to fathom the possible mechanism of action of pesticides with the PPARs [24]. Three main types of cell cultures have been used: those that were transfected, which means that the cell line does not originally express a receptor but is induced [25]; cell lines that express different PPARs, such as the HepG2 line of hepatocytes [26]; and cell lines whose functions depend on activation of the receptor, including the 3T3-L1 cell line of preadipocytes, whose maturation depends on PPAR*γ* [27].  Table 2 shows the reports identified up to this review concerning the different PPARs, the pesticides that activate them, the cell lines used in the studies, and the effect of their activation.

### 3.1. Transactivation of PPARs by Pesticides

To assess the potential biological activity of PPARs through pesticides, two techniques were elaborated; first reporting genes to identify the ability of the complex pesticide-PPAR to bind and translocate to the nucleus cell [25, 28]; and then the recognition and binding to the PPRE via monitoring transcription of regulated genes by the receptors [29]. One of the first analyses of the transactivation of PPAR*α* and PPAR*γ* by the pesticides was carried out in CV-1 cells (kidney cells from monkeys) transfected with the mouse receptors, testing 200 pesticides: 29 organochlorines, 11 diphenyl esters, 59 organophosphates, 12 pyrethroids, 22 carbamates, 11 amides, 7 triazines, 8 ureas, and 44 other groups. The result was that only pyrethrine, imazalil, and diclofob-methyl showed transactivation of PPAR*α*, but none of the 200 pesticides showed transactivation of PPAR*γ*; moreover, these pesticides can activate the RXR, which is also involved in lipid metabolism [25]. These results are consistent with those of 2,4-dichlorophenoxyacetic acid (2,4-D) and 4-chloro-o-toloxyacetic acid (MCPA), previously reported not to transactivate for mouse and human PPAR*α* and PPAR*γ* receptors in transfected COS-1 cells [28]; furthermore, mouse PPAR*α* is more sensitive to the human receptor. Consistent results were observed with pyrethroids: deltamethrin, cis-permethrin, cypermethrin, fenvalerate, allethrin, trans-permethrin, bioresmethrin, and phenothrin, none of which activated the PPAR*α* receptor in transfected COS-1 cells; however, the trans-permethrin metabolites: 3-phenoxybenzoic acid and 3-phenoxybenzaldehyde had agonic activity via PPAR*α* in microarrays [30]. Nevertheless, subsequent studies have shown that many pesticides initially reported as not activators of PPAR*α* and PPAR*γ* can participate in lipid and carbohydrate metabolism, through this receptor as DDT and its metabolite DDE [31], chlorpyrifos [32], diazinon [33], endrin [24], among others.

### 3.2. Activation of PPARs by Pesticides

Thanks to technological progress, it is now possible to obtain and analyze a large amount of data in a short period of time, making it possible to describe in great detail the changes in the genome, proteome, and metabolome at the cell or tissue level in response to toxic environmental influences. The changes induced by the activation of PPARs due to interaction with pesticides have been linked to the development of obesity and type 2 diabetes mellitus [34, 35]. The analysis is mainly based on the findings of differentially expressed genes (DEGs) [36] and transcription factors (TFs) [37], which are the main group of proteins that have a response to the exposure of a chemical substance and specifically increase biological conditions; the result can be associated through networks that allow to identify central and key genes in many diseases [29].

Alteration of PPAR*γ* expression in the presence of toxaphene, methoxychlor, permethrin, atrazine, DDT, paraquat, and chlorpyrifos was described in microarray (RNA) analysis in a rat hepatocyte model, revealing changes in lipid and carbohydrate metabolism [37]. In another analysis of transcriptomics of HepaRG cells and exposure to quizalofop-*p*-ethyl, networks of genes associated with metabolic pathways involved in fatty acid degradation were identified. In the presence of isoxaflutole, retinol metabolism and PPAR*γ* signaling were altered, and finally, glyphosate did not alter the expression of PPAR*γ* but decreased large-chain fatty acids (LCFAs) and polyunsaturated fatty acids (PUFAs), suggesting the existence of other receptors involved in lipid metabolism [29]. In a study using the latest technology proposing organ replacement using an organ-on-chip of a rat kidney, the transcriptome and metabolome were analyzed after DDT and permethrin exposure and their mixture. The results show that the conditions assessed produced a hepatic steatosis profile with high expression of PPAR-related genes, fatty acids, lipid metabolism, and steroid biosynthesis; and the mixture had an additive effect on the transport RNA and necrotic/inflammatory profiles [36].

This omics analysis makes it possible to assign all the changes that can be caused by exposure to pesticides without knowing a possible target for the effects on cellular functions. These results have confirmed to a greater extent the changes found in cell cultures are the expression of specific genes controlled by the PPARs. Therefore, the use of this type of technology can make the detection of changes caused by environmental toxicants more efficiently, including those that were not previously foreseeable. However, the cost and specialized equipment make it difficult to use this tool on a larger scale.

### 3.3. The Biological Effect of PPAR Activation in Lipid Metabolism by the Pesticides

Evaluation of the biological activity of the PPAR*γ* receptor is mainly based on adipocyte differentiation [38], lipid storage in adipose tissue [39], and control of lipid and carbohydrate metabolism [40]. The most commonly used cell line is 3T3-L1 [41]. This strain of preadipocyte is used as a model for initial adipocyte differentiation in assessing activation of the PPAR*γ* receptor [27] and other TFs involved in adipogenesis [42]. However, other cell lines have also been used, such as the OP9 cell line as a model of late adipocyte differentiation [38] and even the use of primary cultures of adipocytes [43]. Less differentiated cells have also been used, such as bone marrow-derived multipotent stromal cells (BM-MSC), which have allowed the evaluation of the role of pesticides on the PPAR*γ* receptor and the other receptors involved in cell differentiation towards the adipocyte lineage [42] and even their possible role in the generation of osteocytes or chondrocytes [38].

To illustrate the biomolecular implications of activation of the PPAR*γ* receptor by pesticides, a brief review of the changes was made and is presented below. The biomarkers used in the preadipocyte cell lines revolve around their differentiation into mature adipocytes. The most important marker is lipid accumulation [27]. Key regulators of adipogenesis that influence and control PPAR*γ* expression include the CCAAT-enhancer-binding protein family (C/EBPs) described as C/EBP*α*, C/EBP*β*, and C/EBP*δ*, with C/EBP*β* being an inducer of C/EBP*α*, which in turn is an inducer of PPAR*γ* [33]; the use of the aP2 gene (fatty acid binding protein) in mature adipocytes is an indicator of its activation [44, 45]. C/EBP*α* and PPAR*γ* promote adipogenesis by controlling the expression of ACC (acetyl-CoA carboxylase), FAS/FASN (fatty acid synthase), FAPB4 (fatty acid binding protein 4), LPL (lipoprotein lipase), which are involved in lipogenesis [42].

However, the expression of proteins is not always sufficient to consider them active, as in the case of ACC, which is controlled by a phosphorylation/dephosphorylation process through AMP-activated protein kinase (AMPK) [23, 44]. On the other hand, the evaluation of CYP4A as an early marker of signaling in peroxisome proliferation has been proposed because it has a PPRE sequence in its promoter [47, 48].

Once adipocytes are mature, other biomarkers are used, such as adipokines, hormones that control adipocyte function are involved in the metabolism of lipids and carbohydrates at local and systemic levels. The adipokines most commonly analyzed in the activation of PPAR*γ* by pesticides are: adiponectin, which is only secreted by mature adipocytes, regulates glucose levels, increases insulin sensitivity, and also has anti-inflammatory effects [33]; leptin, which is directly proportional to adipose tissue [49]; resistin, which regulates insulin sensitivity (in humans by macrophages and in mice by adipocytes) [50]; and perilipin, which plays an important role in the mobilization and accumulation of fat in adipose tissue [33]. Other biomarkers associated with the response to PPAR*γ* activation by pesticides include inflammatory biomarkers: IL-6, monocyte chemotherapy protein 1 (MCP1/CCL2), and tumor necrosis factor-alpha (TNF-*α*), which impair adipocyte differentiation by inhibiting it through the nuclear factor kappa light chain enhancer transcriptional pathway of activating B cells and protein kinase C (NF-/PKC) [51].

Finally, the accumulation of lipids and their subsequent oxidation in mitochondria and peroxisomes lead to high production of reactive oxygen species (ROS), which generate stress in the endoplasmic reticulum (ER) [23] and alter mitochondrial function [40], creating an imbalance in energy homeostasis, factors that have also been studied in adipocyte exposure to pesticides [52].

**Figure 1:**
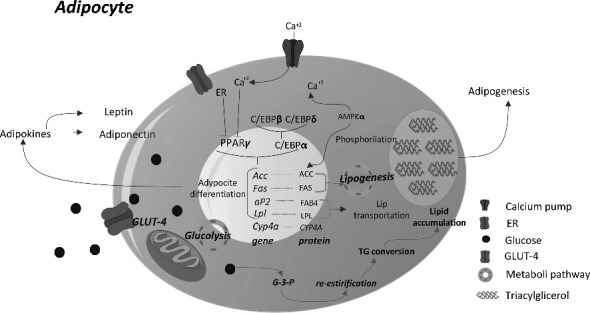
Activation of PPAR*γ* and its interaction with lipid metabolism on the adipocyte. El PPAR*γ* is conditioned to C/EBP*α* activation, once activated the receptor can recognize and bind to a PEP sequence of the genes of *Acc, Fas, Ap2* y *Lpl*, which are involved with lipogenesis, adipocyte differentiation, lipid accumulation, and adipogenesis; lets the secretion of adipokines, therefore, are used as biomarkers of PPAR*γ* activation. Also, the activation can be blocked by the accumulation of Ca^+2^ ions and ER activation. Abbreviations: ACC, acetyl Co-A carboxylase; AMPK*α*, AMP-activated protein kinase; C/EBP*α*-, CCAAT enhancer binding protein alpha; C/EBP-*β*, CCAAT enhancer binding protein beta; C/EBP-d, CCAAT enhancer binding protein delta; ER, estrogen receptor; FABP4, fatty acid-binding protein 4; FAS, fatty acid synthase; GLUT-4, glucose transporter type 4; LPL, lipoprotein lipase; PPAR*γ*, peroxisome proliferator-activated receptor gamma; TG, triglycerides.

It is important to consider the presence and activation of another nuclear steroid/thyroid hormone receptors (NR) associated with adipocyte differentiation and/or function, such as the RXR, which functions by forming a heterodimer with PPARs and influences the processes of cell development, differentiation, metabolism, and death [53]; and the glucocorticoid receptor (GR), which induces adipogenesis and induction of insulin resistance in the mature adipocyte [54].  Figure 1 summarizes the relationships between the various biomarkers mentioned in the adipocyte and provides an overview of the relationships and changes reported by pesticide exposure on adipocyte cellular functioning that affect the development of obesity.

From the group of pesticides belonging to the organochlorines, the effects of DDT and its main metabolite DDE have been studied. Although its use has been banned in several countries, it is still possible to find it in soil samples and various organisms due to the persistence and the accumulation of its metabolite. For DDT, it has been reported to increase the accumulation of lipids, the expression of PPAR*γ* and C/EBP*α* protein, and the enzymes FAS and ACC, and leptin [46, 50]; however, in the 3T3-F442A cell line, leptin levels are increased but C/EBP*α* levels are decreased, possibly leading to late adipocyte differentiation [46]. As for the metabolite DDE, its effect is consistent with that of its parent molecule, as it also increases lipid accumulation, the same enzyme, and adipokines [31] without altering inflammatory markers such as IL-6, MCP-1, and TNF-*α* [50]. Regarding PPAR*α* receptor activation, no difference in mPPAR*α* expression was detected in the 3T3-L1 lineage [49]. Another organochlorine metabolite studied is oxychloride, a metabolite of chlordane, but it has no effect on adipogenesis or lipolysis in NIH3T3-L1 cells [50]. One more, organochlorine pesticide is dieldrin, whose exposure to NIH3T3-L1 cells increases adiponectin and decreases adipogenesis [50].

Among organophosphate pesticides, diazinon induces the accumulation of lipids and increases the expression of de CEBP, FAS, PPAR, ACC, adiponectin, and perilipin, this last one can be found in mature adipocytes [33]. Fenthion is reported to be a PPAR*γ* agonist in both 3T3-L1 and OP9 cell lines and activates the transcriptional activity of PPAR*γ* [38]. Chlorpyrifos was originally reported as an inhibitor of adipocyte differentiation, decreasing lipid accumulation [55], associated with a decrease in leptin, resistin, and adiponectin secretion [49]; but Blanco et al. in 2020 reported an increase in lipid accumulation and increased expression of C/EBP, PPAR, and FAPB4 in the same cell line, 3T3-L1 [32]. On the other hand, the cyclodiene endrin has been reported to inhibit adipogenesis by inhibiting C/EBP [51] and only modestly stimulating PPAR*γ* activity and to a greater extent GR activity [54], so the effect is not associated with PPAR*γ*. However, Seok et al. in 2022 reported that endrin can activate C/EBPs, PPAR*γ*, glucose transporter type 4 (GLUT-4), adiponectin, and FAS in the late phase of adipogenesis [24]. Glyphosate in its commercial form, but not in its pure form, inhibits PPAR*γ* induction, inhibits proliferation and adipogenesis in 3T3-L1; and in mouse embryo fibroblasts (MEFs), it decreases PPAR*γ* but not C/EBP*β*, increases lipid peroxidation and expression of the enzyme superoxide dismutase (SOD) as a process to contain the free radicals and lipids generated during peroxidation [56].

Within the carbamates, methiocarb and carbaryl can activate PPAR*α* [57], while dithiocarbamates such as mancozeb, as antagonists, reduce lipid accumulation and do not affect the expression of either PPAR*α* or PPAR*γ*. As for the imidazoles, prochloraz behaves in the same way as mancozeb as an antagonist [49].

The pyrethroids prallethrin and allethrin have been reported to act as PPAR*γ* agonists to increase the accumulation of lipids in the 3T3-L1 and OP9 lineages, along with suppression of the *Osx* and *Bgalp* genes necessary for osteocyte differentiation into MSC lineages. In addition to increase in FAPB4 levels in the 3T3-L1 lineage following prallethrin exposure [38]. In the case of deltamethrin, it was found to be an antagonist of PPAR*γ* by reducing lipid accumulation and adipocyte differentiation of 3T3-L1 [49].

Quinoxyfen, a member of the quinolines, showed agonistic activity for PPAR*γ* in 3T3-L1 cells; however, it suppressed the expression of osteogenic genes in MSC cells, as did the organotoxic agent fentin [38]. Of the phenylpyrazoles, fipronil increases lipid accumulation and expression of C/EBP, PPAR, CCA, FAS, FABP4, and GLUT-4 [22]. Cis-bifenthrin increases the accumulation of lipids in HepG2 cells and the expression of FAS, PPAR, and SCD1 (stearoyl-CoA desaturase-1), which are responsible for the biosynthesis of monounsaturated fatty acids (MUFAs); however, it has also been shown to do so via the pregnane X receptor (PXR) [58].

The most studied group is the organotin compounds, of which the main representatives are TPT and TBT. TPT is reported to activate PPAR*γ* and RXR, increasing lipid accumulation and adipocyte differentiation [44]. Like TBT, it increases lipids accumulation, activates PPAR*γ*, RXR in its homodimeric form [45], LXR, ER [52], and also increases the expression of the gene aP2, as a marker of adipocyte differentiation [44, 45]. In the multipotent bone marrow stromal cells (BMS2), TBT stimulates lipid accumulation and activates the expression of PPAR*γ*, RXR, and LXR receptors, although the PPAR*γ*-RXR heterodimer is required for the adipogenesis process [59]. In the MSC-C3HI0T1/2 cell line, TBT is able to activate PPAR*γ*2, Pref-1, and Sox9, the latter two genes involved in chondrocyte differentiation. However, the presence of dexamethasone decreases the expression of Pref-1 and SOX9, as well as the gene RUNX2, which is involved in osteocyte differentiation [60]. Regarding primary cultures of adipocytes, there is a report of rainbow trout (*Oncorhynchus mykiss*) adipocytes in which TBT and TPT induce lipid accumulation and increase the expression of PPAR*γ* and C/EBP*α*, but their activation is not sufficient for complete adipocyte differentiation in this species [43].

The phenoxypropidic acid ester quizalofop-*p*-ethyl increases PPAR*γ* expression and lipid accumulation and is a potent inducer of adipogenesis in 3T3-L1. However, the mechanism by which this occurs does not entirely dependent on PPAR*γ* [39]. Chlorantraniliprole, a pyrazole, increases triglyceride content and expression of C/EBP, PPAR, and ACC and decreases pAMPK without altering endoplasmic reticulum stress (ERstress) [23]. Within strobilurins, pyraclostrobin accumulates triglycerides without activating PPAR*γ*, LPL, or C/EBP*α*, so an alternative pathway to that of PPAR*γ* is active, implying a change in mitochondrial function in an attempt by the cell to restore its homeostasis [40].

The study of metabolites derived from pesticides is poorly understood, but for DDE (a metabolite of DDT) in SH-SY5Y cells [31], 3,5,6-trichloropyridinol (TCP) [32] and chlorpyrifos-oxon (CPO) in MCF-7 cells [61], the latter two chlorpyrifos metabolites were reported to have PPAR*γ*-agonizing effects and to promote adipogenesis. The quizalofop-*p*-ethyl metabolites studied (quizalofopic acid, tetrahydrofurfuryl alcohol, and 2,3-dihydroxyquinoxaline) appear to have no activity on adipose tissue [39]. The plasma hydrolysis metabolite of carbaryl, 1-naphthol, is also able to activate PPAR*γ*. However, the hydrolysis metabolite of methiocarb, metylthio-3,5-xylenol, does not activate PPAR*γ* but decreases the expression of PPAR*α* in the presence of the metabolites: methiocarb sulphoxide and methiocarb sulphone [57].

On the other hand, the effect of mixtures of different pesticides is not as researched rather than that of pesticide metabolites, because of the complexity of selecting truly representative mixtures, doses, and the number of pesticides that can be combined. However, the report on mixtures of quizalofop-*p*-ethyl with glyphosate, 2,4-D, dicamba, mesotrione, and isoxaflutole does not appear to have any enhancing or inhibitory effect on its adipogenic effect [29].

The effect of the different pesticides on the PPARs receptors present in or possibly derived from cell lines of the adipocyte lineage shows a great diversity of responses, both agonistic and antagonistic, regardless of the structural similarity between the molecules belonging to the same group of pesticides. Furthermore, the direct effect on the genes activated by the PPARs is very obvious, although it is also recognized that they are not the only nuclear receptors involved in the response; and the final consequences of this alteration in lipid metabolism can also be explained by the change in cellular function of organelles such as the mitochondrial and ER. Given that the mechanism of action of pesticides on PPARs affecting lipid metabolism is very complex and diverse, it is difficult to link pesticides directly to the development of obesity, but this link cannot be denied either.

### 3.4. The Biological Effect of the Activation of PPARs in Carbohydrate Metabolism by Pesticides

The effect of pesticides on the activation of PPARs and carbohydrate metabolism has not been as studied as the liver disturbances in energy metabolism that have been associated to the presence of toxicants. In the literature consulted, only studies concerning the activation of PPARs by pesticides in the HepG2 cell line could be found. Thus, for PPAR*γ*, Ning et al. performed an analysis of 14 pesticides with chitin synthesis inhibitors, 5 of which were found to be potent agonists (diflubenzuron, chlorfluazuron, flucycloxuron, novifluoron, and flufenoxuron). It has been highlighted that diflubenzuron alters energy metabolism by decreasing adenosine triphosphate (ATP) concentrations and increasing those of pyruvate and lactate, two precursor metabolites of the tricarboxylic acid cycle (TAC). The expression of genes encoding enzymes that are part of the TAC such as pyruvate dehydrogenase alpha 1 (PDHA1), oxoglutarate dehydrogenase (OGDH), and citrate synthase (CS) decreases; and with a downward trend in isocitrate dehydrogenase (IDH2) and fumarase (FH), TAC activity decreases. On the other hand, the expression of glycolysis enzymes such as 6-phosphofructo-2-kinase/fructose-2,6-biphosphatase 3 (PFKFB3) and lactate dehydrogenase B (LDHB) is increased. It is possible that these changes favor the synthesis of triglycerides, as glycerol precursors are available in large quantities [20].

In the case of the PPAR*β*/*δ* receptor and its activation by pesticides, a correlation was found between glucose metabolism in HepG2 cells and the herbicide 2,4-D, which lowers extracellular glucose levels and increases glucose in the hepatocyte, associated with increased expression of FoxO1 (increases expression of gluconeogenic genes), CREB (transcriptional regulator of gluconeogenesis), and PPARs [26].

The effect of pesticides on cells more involved in the systemic regulation of carbohydrate metabolism and serum glucose levels is very low, so it is important to conduct further analyses to understand whether the effect has a direct or indirect impact on the development of type 2 diabetes mellitus by inducing insulin resistance and the subsequent development of the disease, which has been raised by different epidemiological studies [9, 62].

### 3.5. Alteration of Lipid and Carbohydrate Metabolism by Activation of PPAR by Pesticides in Other Pathological Conditions

Since the expression of PPARs is diverse in the organs that build up organisms, the effect of their activation not only means a change in carbohydrate and fat metabolism in adipose and liver tissue, which is associated with the development of obesity and diabetes but exposure to pesticides and activation of PPARs has also been shown to be involved in other diseases and even to have a possible protective role in other metabolic processes. The following changes: the cell line used in the study and the observed biological effect are also described in Table 2.

The effect of PPAR*γ* activation by pesticides on tumorigenesis and subsequent cancer development was observed by exposing CD1 mouse, rat, and human hepatocytes to permethrin and its metabolites: 3-phenoxybenzoic acid and trans-dichlorochrysanthemic acid. In the presence of permethrin and 3-phenoxybenzoic acid, DNA replication was increased in mouse cells but not in human cells. In addition to increasing the expression of PPAR*γ* in the presence of 3-phenoxybenzoic acid and trans-dichlorochrysanthemic acid in hepatocytes of mice and rats, but not in humans. There is a clear difference in the response to the activation of the receptor in cells of different species [63].

In human reproductive changes, particularly embryo implantation in the uterus, chlorpyrifos has been reported to be able to damage trophoblast function and placental development in the context of decreasing the expression of PPAR*γ* in an extravillous trophoblast cell model (ecCTB) with HTR8/SVneo cells [64].

The alteration of lipid metabolism in macrophages is influenced by the pesticide TBT, which can activate PPAR*γ*, increase lipid accumulation, expression of lipid metabolism genes in human macrophages (THP-1 cells); such as CD36 (a receptor that promotes the entry of fatty acids into the cell), NR1H3/LXR*α* (regulates the homeostasis of fatty acids and cholesterol), FADS1, FADS2 (catalyze the first step in the synthesis of PUFAs), SREBP-1c (activates hypogenic genes in the liver), ACC (participates in the biosynthesis of fatty acids), FABP4, and FAS [65].

In oxidative stress, PPAR*α* activation may mediate tissue damage due to physical or chemical stress stimuli. Exposure to paraquat increases the presence of CYP4A in primary cultures of mouse hepatocytes of the wild-type genotype and to a greater extent in cells with null PPAR*α*, suggesting regulatory action of PPAR*α* and activation of CYP4A by a different receptor [66].

In the metabolism of lipids in neurons, the metabolite of chlorpyrifos, chlorpyrifos oxon, caused the inhibition of fatty acid amide hydrolase (FAAH), the increase of metabolites of endocannabinoids (eCB), which are agonists of PPARs, as well favored the activation of PPARs in MCF-7 cells and the alteration of lipid metabolism [61].

The activation of PPARs has also been described as a mediator in the damage caused by pesticides that do not activate the receptor or activate it only to a lesser extent, as their effect is abolished by pharmacological agonists of PPARs. PPAR*γ* agonists have been described as dopaminergic neuroprotectors [67], and the most commonly used cell model is SH-SY5Y (human neuroblastoma cells which can differentiate into neurons). Effects of pesticides on this cell line include: deltamethrin decreases the expression of PPAR*γ* and PINK-1 (it is a mitochondrial target involved in protection against ROS) and causes cell death through mitochondria-dependent apoptosis [68]. Chlorpyrifos induces oxidative stress and cell death and also decreases and induces inflammatory genes such as COX-2 and TNF-*α* [69]. Rotenone increases the proliferation of ROS and decreases the expression of SOD1 [70] and TNF-*α* by inhibiting mitochondrial complex I [71]. All these effects are reversed with rosiglitazone as a pharmacological agonist of PPAR*γ*.

Assessment of PPARs receptor activation in cell models provides a guide to understand the molecular mechanism by which the interaction and biological responses achieved in the presence of pesticides occur. However, the information that has been reported up to date is insufficient to generate a general mechanism of action that directly correlates pesticide exposure with the activation of PPARs and the development of obesity. Although the general effect on lipid metabolism, and to a lesser extent carbohydrate metabolism, could be a factor in triggering the development of obesity and, as a complication, the development of diabetes. This is because the reported findings are recurrent. However, several of these results may be due to mechanisms unrelated to the activation of PPARs. Besides, the approach of *in vitro* analyses is limited to a specific cell line, and since the stressful environment might force the cell lines to respond in a way they would not in the presence of other lines, they might mask the responses obtained. Therefore, the use of *in vivo* models may expand the understanding and framing of a systemic response of PPARs receptor activation by pesticides in the development of obesity and type 2 diabetes mellitus.

## 4. *In Vivo* Studies: Activation of PPARs Receptors by Pesticides and Their Subsequent Biological Response

Upstream, animal models have served to evaluate and project the possible effects that might be observed in humans, as what is found in them does not always replicate or approximate the effect observed in humans. In addition, organisms have also been used as sentinel models to assess the degree to which a particular biome is affected by the presence of pesticides or other environmental toxins.  Table 3 shows the animal models used to assess exposure to pesticides involved in PPAR receptor activation and their biological effects.

### 4.1. Changes in Lipid Metabolism in Adipose and Muscle Tissue Involving PPARs due to Pesticides

Lipid metabolism in animal models is assessed by measuring adipose tissue, assessing biomarkers of lipid metabolism in the liver, quantifying triglycerides and cholesterol in serum, and measuring short-chain fatty acids (PFAs) in muscle tissue. Two main animal models were used: aquatic models, which are used to monitor environmental quality, and murine models, which are more focused on clinical implications that can be applied to humans. Then the animal models used to evaluate the activation of PPARs by pesticides involved in lipid metabolism are mentioned.

In the case of triazoles, aquatic animal models are mainly used in the evaluation of their effects, i.e., for paclobutrazol, the rockfish (*Sebasticus marmoratus*) model was used, in which an increase in the expression of PPAR*α* and PPAR*β*/*δ* in the liver and of FAS and ACC1 was observed [72]; for difenoconazole, the marine medaka (*Oryzias melastigma*) model was used, in which an increase in the expression of PPAR*α*, PPPAR*γ*, and PPAR*β*/*δ* was observed in the muscle, but in the liver, only the expression of the first two receptors increased. It is possible that this difference in expression is due to greater oxidation of fatty acids in skeletal muscle tissue and an increase in glucose oxidation in the liver [73].

Glyphosate, in a transcriptomic and proteomic liver analysis of tilapia (*Oreochromis niloticus*), an increase in lipid content but a decrease in the expression of PPAR*α* was observed, this increase in lipids is probably the result of an imbalance in the redox balance of hepatocytes due to a large amount of intracellular ROS [74].

From the group of organotin compounds, TPT decreases the expression of PPAR*γ* and its correlating genes (*Fas, Cyp4b1, Lpl*) in the frog embryo model (*Lithobates sylvaticus*) during the first days of exposure. However, after chronic exposure, this phenomenon reverses and increases the expression of PPAR*α* and PPAR*γ* and related genes; possibly due to an adaptive response to the constant stimulus [75]. Besides, TBT is capable of activating the PPAR*γ* receptor in mouse MSC cells, promoting adipogenesis [76], and increasing adipose tissue mass in adult mice when exposed to the pesticide occurred during mouse fetal development [52]. In the female rat model, it increases adipose tissue weight and increases the accumulation of lipids and cholesterol, as well as the expression of PPAR*γ* and ROS [77].

The use of mouse models to study the activation of PPARs by pesticides can be observed in the analyses performed for mancozeb, a dithiocarbamate, which increased the expression of PPAR*γ* and raised cholesterol and triacylglycerols in the mice serum [78]; however, it was previously reported as an antagonist of PPAR*γ* by not affecting receptor expression and decreasing lipid accumulation in preadipocyte cells [49]. Furthermore, in a mixture of mancozeb and imidacloprid, the increase in cholesterol and triglycerides is enhanced [78]. Including organophosphate, DDT, and DDE could alter adipogenesis processes and reduce PPAR*γ* expression [79]. Another organophosphate, chlorpyrifos promotes obesity but is not related to the expression of PPAR*γ*, it alters mitochondrial function and thermogenesis in mice [7].

Fipronil, an insecticide belonging to the phenylpyrazoles, increased the accumulation of lipids in the liver and altered lipid metabolism by producing an increase in PFAs, which in turn increased the expression of PPAR*α*; and which, when oxidized, increased the concentration of ROS, leading to oxidative stress and activation of inflammatory pathways observed in a rat model [80].

In the case of lambda-cyhalothrin, a pyrethroid capable of activating PPAR*γ* and PPAR*α* receptors in albino rats, it increased the concentration of total lipids, triglycerides, and cholesterol, as well as the inflammatory modulator TNF-*α* [81]. However, Costa et al. demonstrated the differences between pesticides in species and reported that this inflammatory marker did not change in humans in the presence of *α*-cypermethrin, another pyrethroid [82].

Also, the different response of different species to PPAR*γ* activation was evident when evaluating the effect of oxadiazon, an oxadiazole herbicide. In mice and rats, it was observed that exposure to pesticide-induced hepatomegaly due to the enlargement of peroxisomes. However, this effect was not observed in dogs, demonstrating a difference in the sensitivity of PP among species [83].

Dicamba, a salt of benzoic acid used as an herbicide, is a structural isomer of 2,4-D known to be a PP that increases the expression of PPARs and beta-oxidation of lipids, in addition to differential expression of CYP4A with respect to rat sex, as an increase was observed only in males [84]. It is clear that the sex of the organisms can also be considered.

The continuous detection of the change in lipid metabolism and the increase in the expression of PPAR*α* and PPAR*γ* in the presence of different pesticides in the models of aquatic organisms has led to their proposal as biomonitors of water quality and to the possibility of monitoring these changes as biomarkers. The differential response between types of pesticide exposure and their effects on lipid metabolism and PPARs expression allows us to propose a broader study of the characteristics of pesticides and/or organisms that make them more susceptible to pesticide exposure response and the activation of PPARs that favor the alteration of lipids involved in the development of obesity.

### 4.2. Changes in Energy Metabolism in the Liver due to Activation of PPARs by Pesticides

The use of animal models has the advantage that the systemic response to an external stimulus in an organism can be studied. This allows the evaluation of the response of different organs and the compensatory mechanisms of the organism that attempt to minimize and repair the damage caused. Then, various effects of exposure to pesticides on carbohydrate and lipid metabolism as a whole will be described as how both responses relate to the activation of PPARs, as well as their association with the development of obesity and type two diabetes mellitus.

In the organophosphates group, monocrotophos was found to induce glucose intolerance, insulin resistance, and dyslipidemia with hyperinsulinemia in rats, largely due to increased expression of G6FDH (glucose-6-phosphate dehydrogenase) and G3PD (glycerol-3-phosphate), which indirectly promotes the regulation of lipogenesis. The insulin resistance presented is associated with an increase in lipids in the liver, which is favored by increased expression of CCA, FAS, PPAR*γ* (lipogenesis), and a decrease in PPAR*α* (*β*-oxidation). All the previously described changes together produce the symptoms of hepatic steatosis that occur in patients with obesity [85]. On the other hand, chlorpyrifos alters energy metabolism in the liver by decreasing the expression of pyruvate kinase (PK) and glucokinase (GK) enzymes involved in glycolysis, and by decreasing the expression of PPAR*α*, PPAR*γ*, ACO (acyl-CoA oxidase), FAS, ACC (lipid metabolism); in addition to altering the composition of the gut microbiota, decreasing *γ-Proteobacteria*, in the zebrafish (*Danio rerio*) model [86].

Exposure to the organochlorine endosulfan sulfate during pregnancy and early postnatal days in mice resulted in alteration of glucose homeostasis, hepatic lipid metabolism, and gut microbiota; as the expression of PPAR*α*, G6P, GLUT-2 (type 2 glucose transporter) was increased, the opposite effect was observed in the presence of a high-fat diet when biomarkers decreased [87].

Within the group of carbamates, propamocarb has been described to increase GK and decrease PK, PPAR*α*, and genes related to triglyceride and fatty acid synthesis and transport; furthermore, exposure to pesticides has been associated with alteration of the gut microbiota due to alteration of bile acid lipid metabolism, which affects the composition of the microbiota [88, 89]. Another carbamate, carbendazim, which may also be a metabolite of methyl thiophanate and benomyl [90], increases the expression of PPAR*γ*, FAS, hexokinase 1 (HK1) (glycolysis), and PK, in addition to altering the gut microbiota, which decreases the genus *Firmicutes* and *Bacteroidetes*, which is associated with obesity [91]. Thiocarbamate, methyl thiophanate, increases PPAR*α* expression, degrades liver glycogen, and increases ACO, an enzyme involved in lipid metabolism and activation of PPARs, in the Gecko model (*Podarcis sícula*) [92].

In the triazole group, bromuconazole inhibits PPAR*γ* signaling but increases TG, TC, and pyruvate in male rats [18]. Myclobutanil, propiconazole, and triadimefon alter genes regulated by PPAR*α* in male rats, decreasing Cyp4a10, Cyp4a1, and PK, thereby limiting fatty acid biosynthesis and storage [93]. Triazine, a triazide, increases PPAR*β*/*δ* expression and decreases lipid storage. This change may be due to the activation of PPAR*β*/*δ* redirecting metabolism to energy production as an adaptive response to pesticide exposure in frog tadpoles (*Xenopus leavis*) [94].

TBT exposure poses a high risk for the development of type 2 diabetes mellitus in mice because it produces insulin resistance, alters hepatic glucose metabolism, increases insulin levels, and decreases serum glucagon levels. Insulin resistance is caused by increased G6P and phosphoenolpyruvate carboxykinase, which are involved in glycogenolysis and gluconeogenesis [34].

Boscalid, an anilide, affects aquatic organisms such as zebrafish (*Danio rerio*), inhibits their growth and causes liver and kidney damage, increases HK, G6P, and PPAR*α*, promotes *β*-oxidation and decreases ACC, FAS, TG, TC, and blood glucose [95]. Imidacloprid, a neonicotinoid, inhibits zebrafish growth and alters glycolipid metabolism. It increases the expression levels of PPAR*γ*, PPAR*α*, ACC, FAS, GK, and HK, as well as the inflammatory biomarkers TNF-*α*, IL-1*β*, and IL-8, and increases levels in the liver [96].

The above results generally correlate with the increase in lipid storage and lipid oxidation, as well as with the increase in glycogenolysis and gluconeogenesis, and with the development of insulin resistance, which may be the prerequisite for the development of obesity and diabetes. Another factor is the composition of the gut microbiota, which could affect lipid metabolism and promote the development of obesity [91].

### 4.3. Changes in Energy Pathologies Involving Activation of PPARs by Pesticides

Activation of PPARs by pesticides has also been associated with the development of other metabolic disorders or various pathologies, the most obvious of which is associated with altered energy metabolisms, such as obesity and diabetes. Most notably in their role as PP in the development of tumors and liver cancer, in response to oxidative stress, and in response to renal, reproductive, and developmental toxicity.

Several pesticides have been reported to be PP that increase the concentration of peroxisomes in liver tissue and trigger the development of tumors that eventually lead to carcinogenesis. Nuclear receptor activation is a common mechanism of action in the development of toxicity and carcinogenesis in rodents in non-genotoxic processes [97]. Therefore, activation of PPARs by pesticides has been associated with a possible mechanism of carcinogenesis in rodents but not in humans. Pesticides that activate PPARs and have a carcinogenic effect include propaquizafop, an aryloxyphenoxypropionate that has a hepatocarcinogenic effect via PPAR*α* by increasing the expression of CYP4A and ACO in rats but is not relevant to humans according to the Human Relevance Framework (MOA/HRF) [66, 98]. Oxadiazone, an *N*-phenyl heterocycle compound, induces tumor development by activating PPAR*α*, inducing CYPa10 and CYP4A [99]. The benzamide pronamide induces liver tumors through the activation of nuclear receptors CAR (constitutive androstane receptor) and PPAR*α*, which induce genes such as Cyp2b10 and Cy4a10, but is not relevant to the mechanism in humans due to quantitative and qualitative differences between mice and humans [100]. Permethrin, a pyrethroid insecticide, produces liver tumors in mice but not in rats by increasing CYP4A expression and activating PPAR*α*, resulting in a mitotic effect [63], although the effect cannot be extrapolated to humans according to the ILSI/PCS (The International Life Sciences Institute/International Program on Chemical Safety) reference frame. And diclofop, a chlorophenoxy herbicide recognized as PP, increases the number of peroxisomes, palmitoyl-CoA oxidase, catalase, and binucleate hepatocytes in rats, effects that have been associated with PPAR*α* activation [101].

On the other hand, there are pesticides capable of producing tumors, but the involvement of PPARs is not involved in the development of the disease. For example, toxaphene, an organochlorine that causes tumors in the liver of mice, increases genes related to oxidative stress and not PPAR*α* [102]. Within the organophosphate group, methidathione produces tumors in the liver of male mice, but the role of PPAR*α* was ruled out by microarray assay [103]. Finally, there are reports of pesticides suppressing tumor development, such as fenthion, which does not increase liver tumor development, and parathion, which suppresses liver cancer development [103]. And atrazine inhibits cell proliferation and cytokine production in the liver and kidney but does not activate PPARs [104]. Overall, the role of PPAR*α* in tumor/cancer development predominates among pesticides that have been reported to be able to do so by enabling the activation of genes that allow peroxisome proliferation and function. However, it is important to recognize that the receptor is not the only one involved in all pesticides.

Regarding oxidative stress and its relation to obesity, it was observed that lower activity of the PPAR*γ* receptor or its deletion tended to reduce the stress and the expression of obesity. For example, the PPAR*γ*2 Pro12Ala polymorphism decreases its activation and leads to a lower body mass index, and increases insulin sensitivity in humans [105]. Therefore, PPAR*γ*-deficient mice are more resistant to oxidative stress and paraquat exposure lethality, likely due to increased expression of antioxidant genes in adipose and skeletal muscle tissues [105]. However, activation of the PPAR*γ* receptor in lung tissue by agonists such as pioglitazone [106] or carvacrol [107] decreases oxidative stress resulting from paraquat inhalation and, consequently, the production of ROS and inflammatory biomarkers such as TNF-*α* and IL-17, indicating that PPAR*γ* activation elicits a completely different response to the same substance in different organs.

Other effects observed by the activation of PPARS by pesticides include the generation of nephrosis by atrazine and its degradation metabolites, through the activation of PPAR*α* and/or PPAR*γ* receptors, the increase in the expression of CYP4A and its white genes [108]. Testicular toxicity induced by 2,4-D, which is caused by the alteration of PPAR*α* receptor pathways that inhibit cholesterol synthesis in Leydig cells, does not occur in PPAR*α*-null mice [109]. And the induction of malformations, especially in the eye, in frog embryos (*Xenopus tropicallis*) resulting from exposure to TPT due to overexpression of PPAR*γ* [110].

The effect of activation of PPARs by pesticides in pathologies other than obesity and diabetes allows us to highlight that the alteration of lipid metabolism, its intervention in peroxisome proliferation and its differential response in different organs, its evaluation, and analysis of its role in the metabolism of lipids and carbohydrates are more complex than expected.

## 5. Discussion

Based on the above information, it can be concluded that the associations between pesticide exposure and the development of obesity and diabetes mellitus in epidemiological studies have promoted the search for the probable molecular mechanism explaining this relationship. To this end, *in silico* studies were first performed to investigate the interaction between the ligand and the receptor using models such as QSAR or docking. These models revealed that for the PPAR*γ* receptor, the amino acid residue Cys 285 could play a crucial role in the association of pesticides with this receptor, as observed for diflubenzuron [20] or for the organotin compounds TPT and TBT [21]. This interaction was also reported for molecules of a different type such as 1,3-diphenyl-2-propone, volatile organic compounds extracted from a fermented cheonggukjang (Korean food), and the Cys 285 residues of PPAR*β*/*δ* and PPAR*γ* [111]. This interaction was confirmed by X-rays to be essential for the interaction between organotin and the PPARY [21]. The chemical structures of the pesticides that form greater stability in the compound and thus greater activity of the receptor are those that contain aromatic rings in their structure and are able to form *π*–*π* bonds between the amino acid residues that they contain, conforming receptors and their structures, particularly in an ionized state [19, 21], this has also observed with triclocarban, an antiseptic formerly used in personal care products, by interacting with the receptor mouse and human PPAR*α* through a Cl–*π* via the amino acid residue phenylalanine (Phe 318) [112]; in the flame retardants bis(2-ethylhexyl)-2,3,4,5-tetrabromophthalate (TBPH) and mono-2(-ethylhexyl)-tetrabromophthalate (MEHP), where the first *π*-alkyl forms interactions with the amino acid residues histidine (His 199) and proline (Pro 476); and for the second alkyl bonds with the residues methionine (Met 248) and arginine (Arg 260) of the zebrafish PPAR*γ* receptor [113]. This shows that the interaction between the amino acid residues of the receptor and the molecular structures of the pesticides can be given by specific amino acid residues and, moreover, allows specific interactions that enable greater stability.

Since the biological effect of PPAR activation is directly related to the metabolism of lipids and carbohydrates, the main axis of biomarkers studied are the genes containing in their promoter the PPRE and the enzymes/proteins encoded by these genes in the main organs controlling the energetic homeostasis of the organism; the biomarkers used to monitoring PPAR activation through pesticides could be summarized as follows: the biological activation of the PPAR has been observed in adipose tissue by DDT, dieldrin, diazonin, fenthion, and fibronil through the accumulation of lipids as an effect of adipocyte maturation [22, 33, 38, 50]. Also this activation has been observed with organophosphate flame retardants (OPFRs) in inducing adipogenesis as 2-ethylhexyl diphenyl phosphate (EHDPP) [114] and triphenyl phosphate (TPHP) [115]; however, by exploring the mechanism by which pesticides enable this accumulation, it has been shown that the role of PPAR*γ* in this phenomenon is important and consistent, but not unique, as accumulation of lipids is still observed despite blocking the receptor, as in the case of qhizalofop-ethyl [39]; and even the direct role of other receptors in lipid accumulation has been reported, as in the case of dioxin: 2,3,7,8-tetrachlorodibenzo-p-dioxin, which is antagonistic to the aryl hydrocarbon receptor (AhR) and prevents lipid accumulation in association with a decrease in PPAR*γ* [116]. Likewise, the mechanism of lipid accumulation has been shown to be not only due to a process of direct activation of nuclear receptors, as is the case with chlorantraniliprole and pyraclostrobin, which increase oxidative stress in the ER and mitochondria, accordingly, caused by an increase in lipid peroxidation and ROS, and decrease the availability of ATP [23, 40]. In relation to adipogenesis, it has also been observed that the presence of other nuclear receptors is necessary to carry it out, such as RXR, LRX, or ER in organotin: TBT, TPT [45, 52, 59, 60], or the GR for endrin [54], which allows to theorize the existence of a synergistic interaction or enhancer of different receptors by activating a different cascade of regulation and activation of adipogenesis through binding to other receptors also involved in it; for example, TBT forms a covalent bond with the cysteine (Cys) of the active site of the RXR receptor, which activates adipogenesis less efficiently than PPAR*γ* [59]. This could lead to think that the redundant processes present in nature allows to ensure the functioning and maintenance of cells, and even as a regulatory mechanism that overall favors the survival of the organism. In terms of cell differentiation towards the adipocyte lineage, it has been reported that pesticides such as quinoxyfen, fentin, prallethrin, and allethrin show a preference for adipocyte differentiation of MSCs through the PPAR*γ* [38], showing that activation of this receptor is necessary for this outcome by inhibiting genes related to osteocytes or dendrocytes [38].

Antagonists such as deltamethrin, mancozeb, prochloraz, and glyphosate show that complete inhibition of adipogenesis and lipid accumulation corresponds very well with decreased expression of PPAR*γ* and enzymes associated with receptor activation [49, 56]. And that even the effect in the commercial presentation is much greater than in its pure state, as the presence of certain additives can enhance or even increase the activity, as in the case of glyphosate as an antagonist [56] or quizalofop-ethyl as an agonist [39], which has less activity in its pure state. Some additives that have been reported to activate PPAR*α* are toximul [116], a pesticide surfactant, and piperonyl butoxide [118], a pesticide synergist that increases lipid accumulation as well as CYP4A10. Although it is important to highlight that the CYP4A gene contains a PPRE in its promoter, the LXR receptor can also activate this gene [119], revealing an alternative control mechanism and raising the possibility that other genes controlled by PPARs may have a similar mechanism.

In the case of pesticides whose biological activity is controversial because they showed antagonistic activity for PPAR*γ* in initial studies since there is no increase in lipid accumulation, such as chlorpyrifos [49] and endrin [46]; and in later studies, its involvement could be observed by increasing adipogenesis through receptor activation [24, 49, 55]. This discrepancy has been widely discussed and demonstrated in this regard, with results shown to be influenced by the source of strain acquisition, the number of passages in cell culture, and the protocol used during cell differentiation [41]. The protocols used in cell differentiation into adipocytes of the various cell lines use a mixture of a glucocorticoid (dexamethasone), insulin, and 3-isobutyl-1-methylxanthine (MDI), which on the one hand promotes adipogenesis by activating master TFs such as CCAAT-enhancer-binding protein homologous protein (C/EBPs) and PPAR*γ*, and on the other hand, in less differentiated cell lines, inhibits those of other possible lines. In addition to enhancing the stimulus to increase the expression of PPAR*γ*, thereby decanting the differentiation process, as pre-adipocyte cells express low levels of PPAR*γ* [32]. Without the presence of this mixture or any of its components, in the experiments reported, the effect is significantly inhibited or diminished, in addition to obtaining a different pattern of activation and expression of adipogenesis protein. As observed with TBT, the presence of a single element of the MDI mixture favors the expression of adiponectin, perilipin, or C/EBP*α* in a differential manner [120]. Dexamethasone has been described to decrease the expression of adiponectin (which increases insulin sensitivity) and the transcriptional repression of Pref-1 (a key gene in chondrocyte differentiation); insulin favors the activation of PPAR*γ*; and 3-isobutyl-1-methylxanthine increases the concentration of intracellular cAMP, which is necessary for the activation and increase of C/EBP*β* [121]. In addition, the maturity stage of the adipocyte in which the determination is made also affects the observed results. A clear example of this is triterpene, celastrol, which has a stronger effect in the first few days and decreases its effect as it passes through the different stages of maturation [122]. Comparison of results reported to date for pesticides is complicated by the wide variation in methods, which prevents the establishment of a correct mechanism of action for all variants that have an impact. However, it is apparent that they all converge in that activation of PPAR*γ* promotes adipogenesis and lipid accumulation, although the role of the receptor is not yet entirely clear for some pesticides.

The effect of pesticides on PPARs in carbohydrate metabolism has low rates of research, perhaps in large part because it is considered to be the effect of liver injury causing the presence of various toxicants and because the involvement of other receptors in carbohydrate metabolism is more obvious than that of PPARs. However, it has been reiterated that the effect of diflubenzuron on the PPAR*γ* receptor is associated with the decrease of enzymes involved in TCA, reducing their activity and the generation of ATP by this metabolic pathway [20], along with the possibility of increasing the synthesis of triglycerides by increasing the availability of glycerol precursors; and 2,4-D exposure favors the expression of the PPAR*β*/*δ* receptor, allowing the reduction of extracellular glucose and increasing glycogen stores [26]. The previous results highlight the need for more evaluation of the effect of pesticides on carbohydrate metabolism via PPARs. Also, exposure to pesticide mixtures remains controversial due to the absorbability and bioaccumulation of individual pesticides [96].

Although the use of different cell lines has allowed to approach the mechanism by which the activation of PPARs by pesticides may favor the development of obesity and diabetes, the use of *in vivo* models, that is, in animals, has made it possible to analyze the effect of pesticides on several possible target organs simultaneously that were not originally considered and, moreover, to provide a clear approach to the changes that favor the development of disease. The results observed in animal models are very consistent with those observed *in vitro* experiments. Two main models have been used to evaluate the effects of pesticides on PPARs, aquatic models, and murine models. The first model is used more for environmental monitoring, and the second is in the search for understanding diseases and therapeutic targets.

In aquatic models, it was observed that the greatest effect occurs in muscle tissue by increasing the expression of PPAR*α*, PPRA*γ*, and PPAR*β*/*δ* receptors, increasing lipid storage in muscle and adipose tissue, and increasing lipid metabolism, although this effect is not observed in the liver [73]. In murine models, after exposure to pesticides such as TBT, mancozeb, imidacloprid, lambda-cyhalothrin, fipronil, dicamba, and oxadiazon, an increase in PPAR*α* and/or PPAR*γ* receptor exposure is shown in adipose tissue composition, the presence of dyslipidemia (elevated lipids in the blood) and an increase in lipid metabolism associated with an increase in the concentration of ROS and inflammatory biomarkers in adipose tissue, liver, and muscle [80]. In addition, there is a change in the composition of the gut microbiota, which occurs as a direct change in pesticide exposure, as well as a change in fat metabolism and the formation of ROS [86, 89, 91].

However, not all results are in complete agreement with those observed *in vitro* experiments. This was the case with mancozeb [78] and lambda-cyhalothrin [81], where no activation of the receptor or generation of inflammatory biomarkers was observed *in vitro* as shown in the animal model, although it should be noted that the cells and tissues used in the respective experiments are different [82]. Perhaps this is because, in response to toxic exposure, the body attempts to maintain body homeostasis by activating or favoring other receptors that have similar activity to PPARs, such as CAR [72], LXR [72, 73], including estrogen receptors [52], and thyroid receptors [78].

It seems that the alteration of lipid and carbohydrate metabolism because of exposure to pesticides and activation of PPARs is mainly due to an increase in the concentration of lipids, triglycerides, and cholesterol, which are directly related to the increase in the expression of PPAR*α* and PPAR*γ* and the genes activated by these receptors, thus linking them to the development of obesity. On the other hand, the development of insulin resistance is associated with two main processes, the first through the increase in serum lipids and the second through the increase in key enzymes of gluconeogenesis and glycogenolysis (G6P and PKC), leading to an increase in insulin concentration. In a chronic state, insulin resistance develops, leading to the development of type 2 diabetes mellitus.

On the other hand, the recurrently observed increase in oxidative stress and inflammatory biomarkers could be due to the increase of fatty acid in serum, which allows the expression of PPAR receptors, especially in the liver, increasing the accumulation of lipids in this organ, favoring their oxidation, and the consequent production of ROS [77, 80]. The sensitivity of each pesticide to the receptor, the animal species [83], and the genus of the species [84] play an important role in the response obtained during exposure to pesticides.

It is important to highlight that the results presented above link the activation of PPARs receptors in *in vitro* and *in vivo* models to the development of obesity and diabetes, due to the interaction, activation, and biological responses (accumulation of lipids, adipogenesis, alteration in carbohydrates metabolism, insulin resistance); mainly with organotin pesticides (TBT and TPT) [21, 34, 43–45, 59, 76], followed by organophosphates (chlorpyrifos and DDT/DDE) [6, 9, 31, 32, 37, 46, 50, 61, 86]; however, the remaining pesticides such as pyrethroids, carbamates, and others show similar trends in terms of effects on PPARs as initially found for organotin and organophosphates. Therefore, further studies are needed to expand and clarify the mechanism by which pesticides might activate PPARs receptors and cause the development of obesity and diabetes mellitus.

Alteration of the gut microbiota has been raised as a possible target in the development of obesity because alteration of the microbiota has been associated with increased oxidation of lipids and consequent activation of PPARs. The oxidation of lipids and the resulting formation of ROS alter the composition of the microbiota. First, direct exposure to pesticides affects the intestinal mucosa and microbiota composition, favoring the increase of strains associated with the presence of obesity [86, 88, 91].

The mechanism of action by which PP pesticides are capable of producing tumors/cancer has been extensively studied, and the role of PPAR*α* activation appears to be important in this process; even though, the results in rodents are not applicable to humans. It is also recognized that this receptor is not the only one involved in tumorigenesis and that the effect of inhibiting the development of tumors and liver cancer may be the effect of exposure to certain pesticides [97, 103]. Finally, the activation of PPARs has also been suggested as a possible therapeutic target in the cognitive symptoms of Gulf War illness [123].

**Figure 2:**
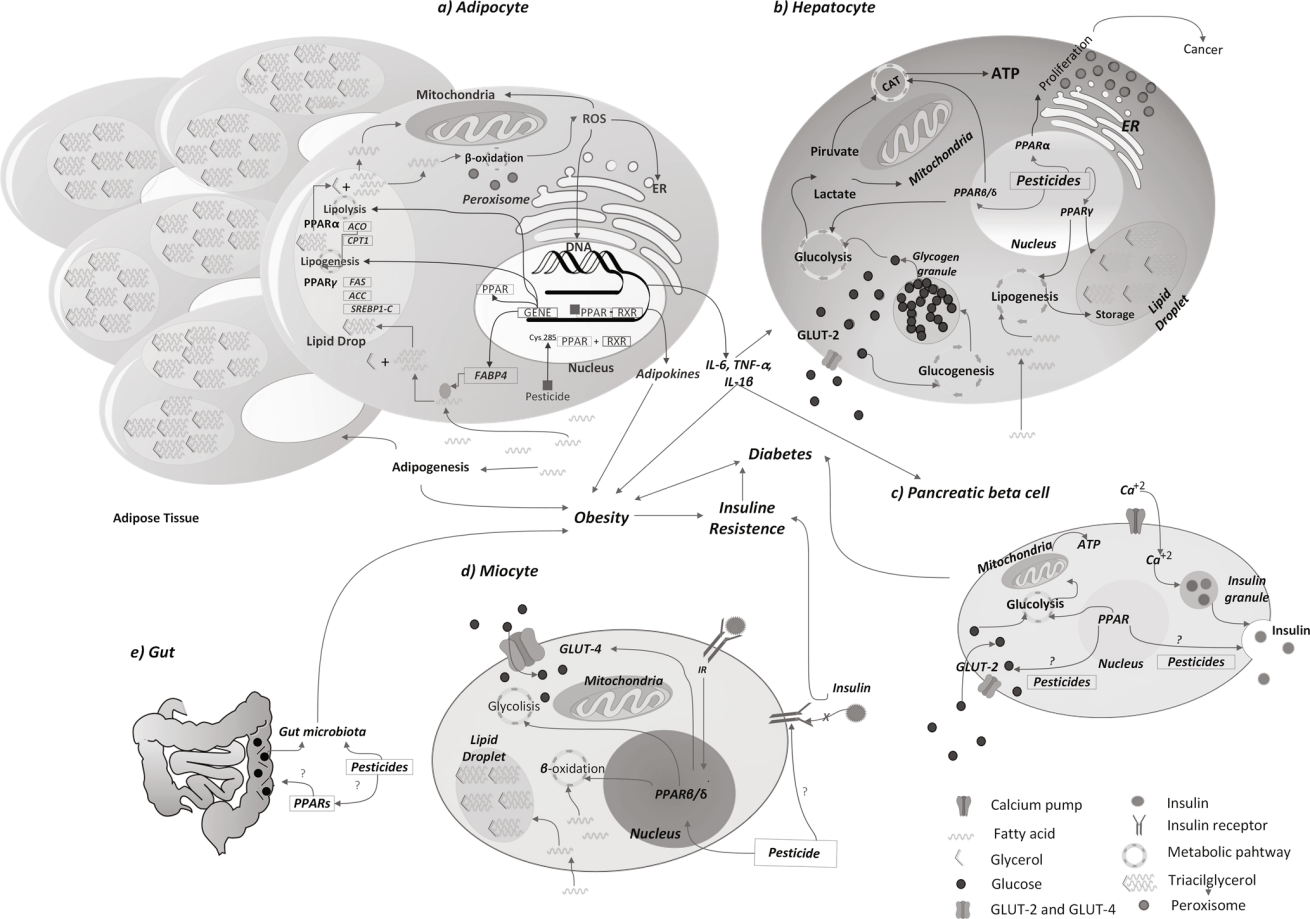
Effects of pesticides on carbohydrate and fat metabolism involving diabetes and obesity through activation of PPARs. Activation of PPARs by pesticides and their effects have been linked to the development of obesity and diabetes in many tissues: adipocytes (a) show mainly PPAR*α* and PPAR*γ* activation to lipid metabolism; in hepatocytes (b) PPAR*γ* is involved in lipid metabolism, PPAR*α* is associated with peroxisome proliferation to cancer and PPAR *β*/*δ* in the carbohydrate metabolism; in pancreatic beta cells (c) the role of PPAR affected by pesticides and the function of these cells is not clear; in myocytes (d) PPAR *β*/*δ* activation is essential for the regulation of energy metabolism, but the effects of pesticide exposure are unclear; finally, (e) the disruption of the gut microbiota in the development of obesity and diabetes about PPAR activation is unknown. Abbreviations: ACC, acetyl Co-A carboxylase; ACO, acyl-CoA oxidase; AMPKa, AMP-activated protein kinase alpha; ATP, adenosine triphosphate; C/EBP-*α*, CCAAT enhancer binding protein alpha; C/EBP-*δ*, CCAAT enhancer binding protein delta; C/EBP-*β*, CCAAT enhancer binding protein beta; CPT-1, carnitine palmitoyltransferase I; ER, endoplasmic reticulum, FABP4, fatty acid-binding protein 4; FAS, fatty acid synthase; GLUT-2, glucose transporter type 2; GLUT-4, glucose transporter type 4; LPL, lipoprotein lipase; PPAR*α*, peroxisome proliferator-activated receptor alpha; PPAR*β*/*δ*, peroxisome proliferator-activated receptor beta/delta; PPAR*γ*, peroxisome proliferator-activated receptor gamma; ROS, reactive oxygen species; RXR, retinoid X receptor; SREBP-1C, sterol regulatory element-binding protein 1, TG, triglycerides.

The above data can be summarized in Figure 2, which shows that the effects on energy metabolism in adipose tissue observed in the presence of pesticides occurred in adipose metabolism through activation of nuclear receptors: PPAR*α*, which controls lipid synthesis genes (ACO and CPT1); and the PPAR*γ* receptor, which controls lipid breakdown genes (FAS, ACC, and SREBP1-C). These, when altered, can trigger the accumulation of ROS (due to lipid degradation), which damages DNA, ER, and mitochondrial function, and promotes oxidative stress: the energy metabolism of the cell is altered. These changes promote the secretion of adipokines and inflammatory components such as adiponectin, leptin, TNF-*α*, and IL-1*β*, which influence and control the entry and consumption of glucose from cells. Lipid metabolism was also altered by the presence of pesticides, increasing the accumulation and growth of adipose tissue and promoting the formation of new adipocytes (adipogenesis). In addition, macrophages present in the tissue were induced to secrete inflammatory components such as TNF-*α*, IL-1*β*, and IL-6. In both cases, a chronic state of inflammation and insulin resistance associated with obesity is promoted. However, since high lipid concentrations prevent the proper functioning of insulin receptors as well as their insufficient synthesis in musculoskeletal tissues, the effect of pesticides at these levels has not yet been studied. On the other hand, changes in energy metabolism were also observed in hepatocytes (Figure 2(b)), in which activation of PPAR*γ* and PPAR*β*/*δ* promotes glycolysis but decreases activation of TAC, thereby increasing the presence of precursors in the metabolism of glycerol, favoring the synthesis of triglycerides, promoting the accumulation of lipids and the development of nonalcoholic fatty liver, and altering systemic energy function. In addition, activation of PPAR*α* promotes peroxisome formation, which has been linked to the development of carcinogenic processes in murine models. The development of diabetes under these conditions may be due to the influence of adipokines (leptin and resistin), as they stimulate or reduce the secretion of insulin (a hormone that controls serum glucose levels) [124]. However, the effects of the presence of pesticides have been little studied and their role is unclear. In contrast, in the pancreas, the organ responsible for insulin secretion (Figure 2(c)), the role of activation of PPARs by pesticides is still unknown, as are the effects on signaling and insulin receptor function in muscle tissue (Figure 2(d)). Finally, alteration of the gut microbiota (Figure 2(e)) appears to alter the metabolism of carbohydrates and lipids; they have recently been proposed as another factor in the development of obesity; nevertheless, the exposure to pesticides at these levels is still unclear. The role of PPARs in the development of obesity and diabetes mellitus is more likely to be the alteration of lipid metabolism and, consequently, the development of insulin tolerance, which in turn triggers the development of type 2 diabetes mellitus, although a greater amount of data is still needed to establish this link.

## 6. Conclusions

According to the WHO, it is estimated that by 2025, approximately 167 millions of people will deal with obesity and problems linked to overweight such as diabetes, high blood pressure, and dyslipidemia. Environmental toxicants, such as pesticides, have been identified as one of the possible factors favoring the development of these diseases; due to their environmental persistence and their bioaccumulation, the exposure to pesticides can take place everywhere: in schools, offices, at home through different vectors such as the food, drinking and daily use water; also traces of pesticides can be found in air, leaving in this way contact to pesticides in all our surroundings. This is why is so important to research and find more info about the effect of these toxicants in the human body and health. Understanding pesticides and their mechanisms of action could allow finding and proposing therapeutic targets to fight them; given that lipid and carbohydrate metabolisms are involved in obesity and diabetes and being that PPARs are one of the most important nuclear receptors for the control of these metabolic processes, we could prevent or avoid activating these interactions.

The information available up to date does not enable to determine the mechanism of action of a specific chemical group of pesticides because compounds in the same group react differently, e.g., as agonists, antagonists, or with no effect on the receptors. The use of *in silico* models has allowed the prediction of the interaction between PPARs and pesticides and has been proven to be a very useful tool.

The use of *in vitro* models with cell lines has been important in elucidating the mechanism of action of pesticides and PPARs, but the discrepancy of the spotted results in different protocols could debate this response. Therefore, the search for alternatives with more precise definitions and lower dispersion of results would allow a better understanding of the effect of pesticides and the mechanism by which they exert this effect.

Finally, *in vivo* models with animals have made it possible to link the effects of pesticides to lipid metabolism and its effects on the development of obesity through the accumulation of fat that ease insulin resistance and the subsequent development of type 2 diabetes mellitus. Thus, activation of PPARs by pesticides was found to be consistent in most cases; PPAR*γ* receptor activation can lead to adipogenesis and lipogenesis in adipose tissue and liver; lipolysis and proliferation of peroxisomes involved in liver tumor development by the PPAR*α* receptor; and changes in lipid and carbohydrate metabolism to a lesser extent by the PPAR*β*/*δ* receptors; however, the changes observed in lipid and even carbohydrate metabolism are not the only ones and are not all exclusively dependent on PPARs, their participation in these metabolic process is essential for the cell function.

The above reviewed statements leave a lot of gaps and opportunities to find more about the alteration of carbohydrate metabolism, and the alteration of pancreatic and muscle-skeleton function associated or not to pesticides, and there are even new implications like the alteration of the gut microbiome associated to the exposure to pesticides and the PPARs leaving many fields in need to be studied with more emphasis.

## Figures and Tables

**Table 1:** *In silico* studies of pesticides and their interaction with PPARs.

PPAR (subtype)	Pesticide	Chemical clasification	Type of pesticide	Analysis	Structure	Item	Software	Year	References
PPAR *γ*	Bromuconazole	Triazole	Fungicide	Docking with the raptors	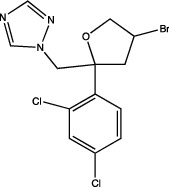	Bind to receptor and act as antagonist	AutoDock Vina	2021	Wu et al. [18]
	Chlorfluazuron	Benzoylurea	Insecticide	Docking with the receptor	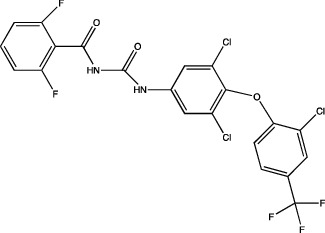	Bind to receptor and act as agonist	Discovery Studio 2.5/LigandFit module	2018	Ning et al. [20]
	Diflubenzuron	Benzoylurea	Insecticide	Docking with the receptor	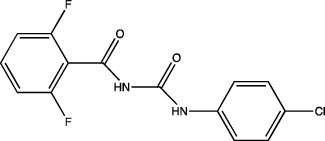	Bind to receptor and act as agonist	Discovery Studio 2.5/LigandFit module	2018	Ning et al. [20]
	Flucycloxuron	Benzoylurea	Insecticide	Docking with the receptor	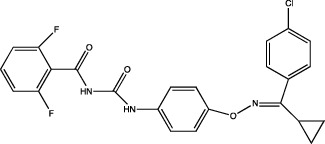	Bind to receptor and act as agonist	Discovery Studio 2.5/LigandFit module	2018	Ning et al. [20]
	Flufenoxuron	Benzoylurea	Insecticide	Docking with the receptor	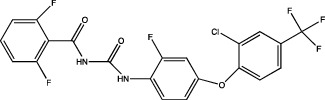	Bind to receptor and act as agonist	Discovery Studio 2.5/LigandFit module	2018	Ning et al. [20]
PPAR *γ*	Noviflumuron	Benzoylurea	Insecticide	Docking with the receptor	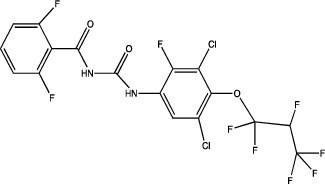	Bind to receptor and act as agonist	Discovery Studio 2.5/LigandFit module	2018	Ning et al. [20]
	Triphenyltin (TPT)	Organotion	Antifouling	X Rays	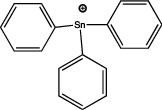	Bind ligand-receptor	MOLREP from the CCP4 suite/Coot and REFMAC5 and MolProbity	2014	Harada et al. [21]
	Tributyltin (TBT)	Organotion	Antifouling	X Rays	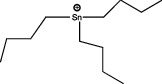	Bind ligand-receptor	MOLREP from the CCP4 suite/Coot and REFMAC5 and MolProbity	2014	Harada et al. [21]
PPAR *γ*	Mancozeb	Dithiocarbamate	Fungicide	Docking with the receptor	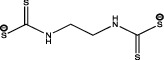	Bind to receptor and act as agonist	Hex Dock and Patch Dock	2014	Bhaskar et al. [78]
	Imidacloprid	Neonicotinoid	Insecticide	Docking with the receptor	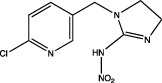	Bind to receptor and act as agonist	Hex Dock and Patch Dock	2014	Bhaskar et al. [78]
PPAR *α*	Fomesafen	Nitrobenzamide	Herbicide	Prediction of bind	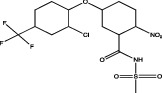	Bind to receptor trough QSAR analysis	Sybyl software suite running on an Evans and Sutherland ESV30	1997	Lewis and Lake [19]

Most of the pesticide structures have a carboxylic group or can form an ion, which let it interact with the residue of aminoacidic of the receptors.

**Table 2:** *In vitro* studies of activation PPARs by pesticides.

PPAR subtype	Pesticide	Chemical classification	Type of pesticide	Cell culture	Item	Year	References
PPAR*α*	Permethrin	Pyrethroid	Insecticide	Primary mouse and human hepatocytes cultures	Activation of PPAR*α*	2020	Kondo et al. [125]
	Methiocarb	Carbamate	Insecticide	Cos-1 cells (transfected)	Activation of PPAR*α*, CYP4A, PXR, CAR	2016	Fujino et al. [57]
	Carbaryl				Activation of PPAR*α*, PXR, CAR		
	Deltamethrin	Pyrethroid	Insecticide	Cos-1 cells (transfected)	None activate PPAR*α*	2019	Fujino et al. [30]
	Cis-Permethrin Cypermethrin						
	Paraquat	Bipyridine	Herbicide	Primary mouse hepatocytes cultures	Activation of PPAR*α*, regulate lipid homeostasis and dismiss of stress	2004	Anderson et al. [66]
PPAR *β*/*δ*	2,4-D	Phenoxy	Herbicide	HepG2 cell line	Increase expres of PPAR*β*/*δ*, and CREB (regulator of gluconeogenesis)	2018	Sun et al. [26]
PPAR*γ*	Endrin	Organochlorine	Insecticide	3T3-L1 cell line	Up-regulate PPAR*γ*, C/EBPs, FAS, GLUT-4, Adiponectin	2022	Seok et al. [24]
	DDT	Organochlorine	Insecticide	Primary hepatocytes rat	Increase PPAR*γ*	2021	Jellali et al. [36]
	Permethrin	Pyrethroid					
	Chlorpyrifos	Organophosphate	Insecticide	3T3-L1 cell line	Enhance store lipid droplets, up-regulated transcription of PPAR*γ*, C/EBP*α* and FABP4	2020	Blanco et al. [32]
	Permethrin	Pyrethroid	Insecticide	3T3-L1 cell line	TG accumulation and pre-adipocytes proliferation	2021	Kassotis et al. [126]
	Cypermethrin						
	Chlorpyrifos						
		Organophosphate					
	Iprodione	Imide		Hepatocyte rat cell line	Up-regulate PPAR*γ*	2020	Sohrabi et al. [37]
			Fungicide				
	Flutolanil	Acid amides	Fungicide				
	Paraquat	Bipyridine	Herbicide				
	DDT	Organochlorine	Insecticide				
	Endosulfan		Insecticide				
	Methoxychlor		Insecticide				
	Pentachlorophenol		Insecticide				
	Quintozene		Fungicide				
	Toxaphene		Insecticide				
	Chlorpyrifos	Organophosphate	Insecticide				
	Diazinon		Insecticide				
	Fibronil	Phenylpyrazole	Fungicide				
	Allethrin	Pyrethroid	Insecticide				
	Bifenthrin		Fungicide				
	Cyhalothrin		Insecticide				
	Permethrin		Insecticide				
	Resmethrin		Insecticide				
	Atrazine	Triazine	Herbicide				
	Paclobutrazol	Triazole	Herbicide				
	Diuron	Ureas	Herbicide				
	Endrin	Organochlorine	Insecticide	Hepatocyte rat cell line	Down-regulate PPAR*γ*	2020	Sohrabi et al. [37]
	Propamocarb	Carbamate	Fungicide				
	Rotenone	Heteropentacyclic compound	Insecticide				
	Prallethrin	Pyrethroid	Insecticide	3T3-L1, OP9, BM-MSC cell lines	Lipid accumulation, activate PPAR*γ* and stimulate the expression of Plin1	2020	Andrews et al. [38]
	Allethrin	Pyrethroid	Insecticide	3T3-L1, OP9, BM-MSC cell lines	Activate transcriptional function of PPAR*γ*	2020	Andrews et al. [38]
	Fenthion	Organophosphate	Insecticide				
	Fentin	Organotion	Fungicide				
	Quinoxyfen	Quinoline	Fungicide				
	2-Benzothiazole sulfonic acid	Benzothiazole	Fungicide	Mammalian cells	Bind to PPAR*γ*	2020	Neale et. al. [127]
	MCPA	Phenoxy	Herbicide				
	TBT	Organotion	Antifouling	THP-1cell line	Activation of PPAR*γ*, increase lipid accumulation and expression of lipid metabolism genes	2021	Jie et al. [65]
	QpE	Phenoxy	Herbicide	3T3-L1 cell line	Induce accumulation of lipids via PPAR*γ*	2019	Biserni et al. [39]
	Glyphosate	Organophosphate	Herbicide	3T3-L1 cell line	Not induce accumulation of lipids via PPAR*γ*	2018	Mesnage et al. [29]
	2,4-D	Phenoxy	Herbicide				
	Dicamba	Chlorophenoxy	Herbicide				
	Mesotrione	Triketone	Herbicide				
	Isoxaflutole	Isoxazole	Herbicide				
	Permethrin	Pyrethroid	Insecticide	3T3-L1 cell line	Induce adipogenesis via PPAR*γ*	2019	Qi et al. [128]
	Fibronil	Phenylpyrazole	Fungicide				
	Chlorantraniliprole	Ryanoid	Insecticide	3T3-L1 cell line	Induce adipogenesis, up-regulate C/EBP*α*, PPAR*γ* and ACC	2019	Yuan et al. [23]
	Chlorpyrifos	Organophosphate	Insecticide	HTR8/SVneo cells	Reduce mRNA of PPAR*γ*	2019	Ridano et al. [64]
	Flubendiamide	Ryanoid	Insecticide	3T3-L1 cell line	Enhance TG content, increase C/EBP*α*, PPAR*γ*	2018	Sun et al. [129]
	Pyraclostrobin	Strobilurin	Fungicide	3T3-L1 cell line	TG accumulation, without activation of PPAR*γ*, reduce LPL, CEBP*α*, GLUT4	2018	Luz et al. [40]
	Cis-Bifenthrin	Pyrethroid	Insecticide	HepG2 cell line	Lipid accumulation, induce expression of PPAR*γ*, FAS	2018	Xiang et al. [58]
	QpE	Phenoxy	Herbicide	HepaRG (transfected)	Activate PPAR*γ*	2018	Mesnage et al. [37]
	Isoxaflutole	Isoxazole	Herbicide				
	Mesotrones	Triketone	Herbicide				
	Glyphosate	Organophosphate	Herbicide		Not activate PPAR*γ*		
	Diazinon	Organophosphate	Insecticide	3T3-L1 cell line	Increase lipid accumulation, induce transcriptional factors of C/EBP*α* and PPAR*γ*	2018	Smith et al. [33]
	Diflubenzuron Chlorfluazuron Flucycloxuron Noviflumuron	Benzoylurea	Insecticide	HepG2 cell line	Exhibited potent PPAR*γ* agonistic activity	2018	Ning et al. [20]
	Flufenoxuron						
	TBT	Organotion	Antifouling	Primary adipocytes culture of *Onchrorynchus mykiss*	Induce lipid accumulation, increase C/EBP*α* and PPAR*γ* expression	2017	Lutfi et al. [43]
	TPT						
	DDT / DDE	Organochlorine	Insecticide	3T3-L1 cell line	Increase lipid accumulation, PPAR*γ* expression, FAS, C/EBP*α*, LPL	2016	Kim et al. [31]
	Fibronil	Phenylpyrazole	Insecticide	3T3-L1 cell line	Increase lipid accumulation and expression of PPAR*γ* and C/EBP*α* genes	2016	Sun et al. [22]
	Glyphosate	Organophosphate	Herbicide	3T3-L1 cell line	Increase lipid peroxidation, inhibit the induction of PPAR*γ* during differentiation with the commercial presentation, not in pure form	2016	Martini et al. [56]
	Deltamethrin	Pyrethroid	Insecticide	SH-SY5Y cell line	Decreased PPAR*γ* expression and the receptor protects against pesticide cytotoxicity	2016	Ko et al. [68]
	TBT	Organotion	Antifouling	MSC cells	Promote adipogenesis via PPAR*γ* receptor but there are others receptors	2014	Biemann et al. [60]
	Chlorpyrifos	Organophosphate	Insecticide	SH-SY5Y cell line	Activation of PPAR*γ*, dismiss the oxidative stress, inflammation and death cell produced to the pesticide	2014	Lee et al. [69]
	Rotenone	Heteropentacyclic compound	Insecticide	SH-SY5Y cell line	Activation of PPAR*γ* via rosiglitazone and inhibits the effect of pesticide	2014	Corona et al. [70]
	TBT	Organotion	Antifouling	MSC cells	Induce PPAR*γ*, FABP4, lipid accumulation, stimulus cellular differentiation	2011	Yanik et al. [59]
	TPT						
	Dibutyltin						
	TBT	Organotion	Antifouling	3T3-L1 cell line	Increase adipogenic activity but not via PPAR*γ*	2011	Penza et al. [52]
	Endrin	Organochlorine	Insecticide	3T3-L1 cell line	Bind to PPAR*γ*, but preferably through to glucocorticoid receptor	2010	Sargis et al. [54]
	Tolylfluanid	Sulfamide	Fungicide				
	TPT	Organotion	Antifouling		Activate PPAR*γ*		
	TBT	Organotion	Antifouling	3T3-L1 cell line	Promote adipogenesis and lipid accumulation	2006	Grün et al. [130]
	TBT	Organotion	Antifouling	3T3-L1 cell line	Accumulation of lipid but not via PPAR*γ* and increase aP2	2005	Inadera & Shimomura [45]
	TBT	Organotion	Antifouling	3T3-L1 cell line	Activate PPAR*γ*, accumulation of TG and increase adipocyte differentiation	2005	Kanayama et al. [44]
	TPT						
	DDT	Organochlorine	Insecticide	3T3-L1 cell line	Induction of C/EBP*α*, PPAR*γ*, increase phenotype of adipocytes	2002	Moreno-Aliaga & Matsumura [46]
	Endrin	Organochlorine	Insecticide	3T3-L1 cell line	Inhibition of adipocyte differentiation, inhibit C/EBP*α* but not C/EBP*β* and C/EBP*δ*, reduce PPAR*γ*	1999	Moreno-Aliaga & Matsumura [51]
PPAR*α* and PPAR*γ*	DDE	Organochlorine	Insecticide	3T3-L1 cell line	No affect PPAR*α* neither PPAR*γ*, reduce lipid accumulation inhibit adipocyte differentiation	2012	Taxvig et al. [49]
	Chlorpyrifos	Organophosphate	Insecticide				
	Mancozeb	Dithiocarbamate	Fungicide				
	Prochoraz	Ureas	Fungicide				
	Deltamethrin	Pyrethroid	Insecticide	3T3-L1 cell line	Activate PPAR*γ* but not PPAR*α*, reduce lipid accumulation	2012	Taxvig et al. [49]
	Aldrin	Organochlorine	Insecticide	CV-1 cell line transfected with PPAR*α* and PPAR*γ* mouse			
	*α*-BHC		Insecticide				
	*β*-BHC		Insecticide				
	*γ*-BHC		Insecticide				
	*δ*-BHC		Insecticide				
	Captan		Fungicide				
	cis-Chlordane		Insecticide				
	trans-Chlordane		Insecticide				
	Chlorobenzilate		Insecticide				
	Chloropropylate		Insecticide				
	Chlorothalonil		Fungicide				
	o,p´-DDT		Insecticide				
	p,p´-DDT		Insecticide				
	p,p´-DDE		Insecticide				
	p,p´-DDD		Insecticide		None have agonistic activity to PPAR*α* and PPAR*γ*	2006	Takeuchi et al. [25]
	Dichlobenil		Herbicide				
	Dicofol		Insecticide				
	Dieldrin		Insecticide				
	*α*-Endosulfan		Insecticide				
	*β*-Endosulfan		Insecticide				
	Endosulfan sulfate		Insecticide				
	Endrin		Insecticide				
	Folpet		Fungicide				
	Fthalide		Fungicide				
	Heptachlor		Insecticide				
	Heptachlor epoxide		Insecticide				
	Methoxychlor		Insecticide				
	Pentachlorophenol		Insecticide				
	Quintozene		Fungicide				
	Acifluorfen	Diphenyl ethers	Herbicide	CV-1 cell line transfected with PPAR*α* and PPAR*γ* mouse	None have agonistic activity to PPAR*α* and PPAR*γ*	2006	Takeuchi et al. [25]
	Acifluorfen-methyl		Herbicide				
	Bifenox		Herbicide				
	Chlomethoxyfen		Herbicide				
	Chlornitrofen		Herbicide				
	CNP-amino		Herbicide				
	Chloroxurone		Herbicide				
	Diclofop-methyl		Herbicide	CV-1 cell line transfected with PPAR*α* and PPAR*γ* mouse	Induce PPAR*α* and PPAR*γ*	2006	Takeuchi et al. [25]
	Fluazifop-butyl		Herbicide	CV-1 cell line transfected with PPAR*α* and PPAR*γ* mouse	None have agonistic activity to PPAR*α* and PPAR*γ*	2006	Takeuchi et al. [25]
	Nitrofen		Herbicide				
	Oxyfluorfen		Herbicide				
	Acephate	Organophosphate	Insecticide				
			
			
			
			
			
			
			
			
				CV-1 cell line transfected with PPAR*α* and PPAR*γ* mouse	None have agonistic activity to PPAR*α* and PPAR*γ*	2006	Takeuchi et al. [25]
			
	Anilofos		Herbicide				
	Bromophos-ethyl		Insecticide				
	Bromophos-methyl		Insecticide				
	Butamifos		Herbicide				
	Chlorpyrifos		Insecticide				
	Chlorpyrifos-methyl		Insecticide				
	Cyanofenphos		Insecticide				
	Cyanophos		Insecticide				
	Diazinon		Insecticide				
	Dichlofenthion		Insecticide				
	Dichlorvos		Insecticide				
	Dimethoate		Insecticide				
	Dioxabenzofos		Insecticide				
	Disulfoton		Insecticide				
	EPN		Insecticide				
	Edifenphos		Fungicide				
	Ethion		Insecticide				
	Ethoprophos		Insecticide				
	Fenamiphos		Nematicide				
	Fenchlorphos		Insecticide				
	Fenitrothion		Insecticide				
	Fenitrothion oxon		Insecticide				
	Fensulfothion		Insecticide				
	Fenthion		Insecticide				
	Glyphosate		Herbicide				
	Iprobenfos		Fungicide				
	Isofenphos		Insecticide	CV-1 cell line transfected with PPAR*α* and PPAR*γ* mouse	None have agonistic activity to PPAR*α* and PPAR*γ*	2006	Takeuchi et al. [25]
	Isoxathion		Insecticide				
	Leptophos		Insecticide				
	Malathion		Insecticide				
	Mecarbam		Insecticide				
	Methamidophos		Insecticide				
	Methidathion		Insecticide				
	Methyl-parathion		Insecticide				
	Monocrotophos		Insecticide				
	Parathion		Insecticide				
	Phenthoate		Insecticide				
	Phorate		Insecticide				
	Phosalone		Insecticide				
	Phosmet		Insecticide				
	Piperophos		Fungicide				
	Pirimiphos-methyl		Insecticide				
	Profenofos		Insecticide				
	Propaphos		Insecticide				
	Prothiofos		Insecticide				
	Prothiofos oxon		Insecticide				
	Pyridaphenthion		Insecticide				
	Quinalphos		Insecticide	CV-1 cell line transfected with PPAR*α* and PPAR*γ* mouse	None have agonistic activity to PPAR*α* and PPAR*γ*	2006	Takeuchi et al. [25]
	Terbufos		Insecticide				
	Tetrachlorvinphos		Insecticide				
	Thiometon		Insecticide				
	Tolclofos-methyl		Fungicide				
	Tolclofos-methyl oxon		Fungicide				
	Trichlorfon		Insecticide				
	Vamidothion		Insecticide				
	Cyfluthrin	Pyrethroid	Insecticide	CV-1 cell line transfected with PPAR*α* and PPAR*γ* mouse	None have agonistic activity to PPAR*α* and PPAR*γ*	2006	Takeuchi et al. [25]
	Cyhalothrin		Insecticide				
	Cypermethrin		Insecticide				
	Deltamethrin		Insecticide				
	Etofenprox		Insecticide				
	Fenvalerate		Insecticide				
	Flucythrinate		Insecticide				
	Fluvalinate		Insecticide				
	Permethrin		Insecticide				
	Pyrethrin		Insecticide	CV-1 cell line transfected with PPAR*α* and PPAR*γ* mouse	Induce PPAR*α* and PPAR*γ*	2006	Takeuchi et al. [25]
	Tefluthrin		Insecticide	CV-1 cell line transfected with PPAR*α* and PPAR*γ* mouse	None have agonistic activity to PPAR*α* and PPAR*γ*	2006	Takeuchi et al. [25]
	Tralomethrin		Insecticide				
	Bendiocarb	Carbamate	Insecticide	CV-1 cell line transfected with PPAR*α* and PPAR*γ* mouse	None have agonistic activity to PPAR*α* and PPAR*γ*	2006	Takeuchi et al. [25]
	Benomyl		Fungicide				
	Carbaryl		Insecticide				
	Carbendazim		Fungicide				
	Carbofuran		Insecticide				
	Chlorpropham		Herbicide				
	Diethofencarb		Fungicide				
	Dimepiperate		Herbicide				
	Esprocarb		Herbicide				
	Ethiofencarb		Insecticide				
	Fenobucarb		Insecticide				
	Isoprocarb		Insecticide				
	Methiocarb		Insecticide				
	Methomyl		Insecticide				
	Molinate		Herbicide				
	Oxamyl		Insecticide				
	Phenmedipham		Herbicide				
	Pirimicarb		Insecticide				
	Pyributicarb		Herbicide				
	Thiobencarb		Herbicide				
	Thiobencarb sulfon		Herbicide				
	Thiram		Fungicide				
	Alachlor	Acid amides	Herbicide	CV-1 cell line transfected with PPAR*α* and PPAR*γ* mouse	None have agonistic activity to PPAR*α* and PPAR*γ*	2006	Takeuchi et al. [25]
	Asulam		Herbicide				
	Cafenstrole		Herbicide				
	Flutolanil		Fungicide				
	Mefenacet		Herbicide				
	Mepronil		Fungicide				
	Metalaxyl		Fungicide				
	Metolachlor		Herbicide				
	Pretilachlor		Herbicide				
	Propyzamide		Herbicide				
	Thenylchlor		Herbicide				
	Anilazine	Triazine	Fungicide	CV-1 cell line transfected with PPAR*α* and PPAR*γ* mouse	None have agonistic activity to PPAR*α* and PPAR*γ*	2006	Takeuchi et al. [25]
	Atrazine		Herbicide				
	Metribuzin		Herbicide				
	Prometon		Herbicide				
	Prometryn		Herbicide				
	Simazine		Herbicide				
	Simetryn		Herbicide				
	Bensulfuron-methyl	Ureas	Herbicide				
	Daimuron		Herbicide				
	Diflubenzuron		Insecticide				
	Diuron		Herbicide				
	Linuron		Herbicide				
	Pencycuron		Fungicide				
	Prochloraz		Fungicide	CV-1 cell line transfected with PPAR*α* and PPAR*γ* mouse	None have agonistic activity to PPAR*α* and PPAR*γ*	2006	Takeuchi et al. [25]
	Propanil		Herbicide				
	Amitraz	Formamidine	Fungicide	CV-1 cell line transfected with PPAR*α* and PPAR*γ* mouse	None have agonistic activity to PPAR*α* and PPAR*γ*	2006	Takeuchi et al. [25]
	Benfuresate	Benzofuran	Herbicide				
	Bentazone	Benzothiadiazole	Herbicide				
	Benzoximate	Organochlorine	Acaricide				
	Bitertanol	Triazole	Fungicide				
	Bromopropylate	Benzilate	Acaricide				
	Chinomethionat	Quinoxaline	Fungicide				
	Chloridazon	Pyridazinone	Herbicide				
	Dazomet	Thiadiazine	Insecticide				
	Diquat	Bipyridine	Herbicide				
	Fenarimol	Pyrimidine	Fungicide				
	Ferimzone	Pyrimidine	Fungicide				
	Fluazinam	Diarilamine	Fungicide				
	Imazalil	Conazole	Fungicide	CV-1 cell line transfected with PPAR*α* and PPAR*γ* mouse	Induce PPAR*α* and PPAR*γ*	2006	Takeuchi et al. [25]
	Imidacloprid	Neonicotinoid	Insecticide				
	Iminoctadine	Guanidine	Fungicide				
	Indanofan	Sulfonylurea	Herbicide				
	Ioxynil	Nitrile	Herbicide	CV-1 cell line transfected with PPAR*α* and PPAR*γ* mouse	None have agonistic activity to PPAR*α* and PPAR*γ*	2006	Takeuchi et al. [25]
	Iprodione	Imide	Fungicide				
	Isoprothiolane	Dithiolane	Fungicide				
	Lenacil	Uracyles	Herbicide				
	MCPA	Phenoxy	Herbicide				
	2,4-D	Phenoxy	Herbicide				
	Paraquat	Bipyridine	Herbicide				
	Pendimethalin	Dinitroaniline	Herbicide				
	Probenazole	Benoxthiazole	Fungicide				
	Procymidone	Dicarboximide	Fungicide				
	Propiconazole	Triazole	Fungicide				
	Pyrazolynate	Pyrazole	Herbicide				
	Pyrazoxyfen	Pyrazole	Herbicide				
	Pyroquilon	Pyrroloquinoline	Fungicide	CV-1 cell line transfected with PPAR*α* and PPAR*γ* mouse	None have agonistic activity to PPAR*α* and PPAR*γ*	2006	Takeuchi et al. [25]
	Sethoxydim	Oxime	Herbicide				
	Thiabendazole	Benzimidazole	Fungicide				
	Thiocyclam	Nereistoxin	Insecticide				
	Thiophanate-methyl	Thioureas	Fungicide				
	Triadimefon	Triazole	Fungicide				
	Tricyclazole	Triazole	Fungicide				
	Triflumizole	Imidazole	Fungicide				
	Trifluralin	Dinitroaniline	Herbicide				
	Triforine	Piperazine	Fungicide				
	Vinclozolin	Dicarboximide	Fungicide				
	2,4-D MCPA	Phenoxy	Herbicide	COS-1 (transfected)	No transactivation receptors	1999	Maloney & Waxman [28]
PPAR*α*, PPAR*β*/*δ*, PPAR*γ*	Atrazine	Triazine	Herbicide	RK13 (rabbit kidney) transfected	No interaction with the receptors	2003	Devos et al. [104]

Abbreviations: 2,4-D, 2,4-dichlorphenoxyacetic acid; ACC, acetyl Co-A carboxylase; BM-MSC, bone marrow-derived mesenchymal stem cell; C/EBP*α*, CCAAT enhancer binding protein alpha; C/EBP*β*, CCAAT enhancer binding protein beta; CAR, constitutive androstane receptor; CREB, CAMP responsive element binding protein 1; EPN, Ethyl p-nitrophenyl phenylphosphorothioate; FABP4, fatty acid-binding protein 4; FAS, fatty acid synthase; GLUT-4, glucose transporter type 4; LPL, lipoprotein lipase; MCPA, 4-Chloro-o-toloxyacetic acid; DDT, diclorodifeniltricloroetano; DDD, Dichlorodiphenyldichloroethane; DDE, Dichlorodiphenyldichloroethylene; PPAR*α*, peroxisome proliferator-activated receptor alpha; PPAR*β*/*δ*, peroxisome proliferator-activated receptor beta or delta; PPAR*γ*, peroxisome proliferator-activated receptor gamma; PXR, pregnane X receptor; QpE, Quizalofop-p-ethyl; TBT, Tributyltin; TG, triglycerides; TPT, Triphenyltin.

**Table 3:** *In vivo* models of pesticide effect above the PPAR receptors.

PPAR subtype	Pesticide	Chemical classification	Type of pesticide	Model of study	Item	Year	References
PPAR*α*	Methidathion	Organophosphate	Insecticide	Male B6C3F1 mice	These pesticides not active PPAR*α* in a tumorigenesis process	2022	Rooney et al. [103]
	Fenthion						
	Parathion						
	Fibronil	Phenylpyrazole	Insecticide	Male albino rats	Up-regulated FABP, ACC1, and PPAR*α*	2021	Wasef et al. [80]
	Carbendazim	Carbamate	Fungicide	Male zebrafish (*Danio rerio*)	Level of glucose decreased and PPAR*α*, ACO, CPT1 were not affected	2020	Bao et al. [91]
	Boscalid	Anilide	Fungicide	Zebrafish (*Danio rerio*)	Decrease the content of TG and cholesterol by accelerating lipolysis; and inhibiting lipogenesis, via the regulation of PPAR*α*	2019	Qian et al. [95]
	Permethrin	Pyrethroid	Insecticide	Female C57BL/6N wild-type or PPAR*α* (KO) mice	Increase expression of PPAR*α* in hepatocytes and KO mice the effect decreases	2019	Kondo et al. [63]
	Propaquizafop	Ariloxiphenoxypropionate	Herbicide	Male SD wild-type or PPAR*α* (KO) rats	PPAR*α* regulates the biochemical and histological changes in the liver in hepatocarcinogenesis	2018	Strupp et al. [98]
	Propamocarb	Carbamate	Fungicide	Male C57bL/6J mice	Decrease PPAR*α* and increase hepatic bile acids with a change of energy metabolism and the gut microbiota	2018	Wu et al.^89^
	2,4-D	Organochlorine	Herbicide	Male Sv/129 wild-type or PPAR*α*-null mice	Induce testicular toxicity due to disruption of cholesterol/testosterone homeostasis in Leydig cells via PPAR*α*	2016	Harada et al. [109]
	Oxadiazon	Oxadiazol	Herbicide	Male C3H/HeNCrl and CAR (KO) mice	PPAR*α* and CAR are involved in the development of liver tumors	2016	Kuwata et al. [99]
	Toxaphene	Organochlorine	Insecticide	Male B6C3F1 mice	Induce mouse liver tumors, increase CAR, AhR but not PPAR*α* target genes	2015	Wan et al. [102]
	Myclobutanil	Triazole	Fungicide	Male Wistar Han IGS rats	Perturb fatty acid and steroid metabolism in the liver predominantly through the CAR, PPAR*α*, and PXR signaling pathways.	2009	Goetz and Dix [93]
	Propiconazole						
	Triadimefon						
	Methyl thiophanate	Thioallophanate	Fungicide	Male lizard (*Podarcis sicula*)	Increase AOX and PPAR*α*	2006	Buono et al. [92]
PPAR*β*/*δ*	Atrazine	Triazine	Herbicide	*Xenopus leavis* tadpoles	Increase PPAR*β*/*δ*, which is associated with the conversion of lipid and proteins into energy	2011	Zaya et al. [94]
PPAR*γ*	DDT	Organophosphate	Insecticide	Male SD rats	Decrease PPAR*γ* expression	2022	Al-Obaidi [79]
	DDE						
	Bromuconazole	Triazole	Fungicide	Male SD rats	Decrease the TG synthesis via inhibiting the PPAR*γ* pathway	2021	Wu et al. [18]
	TBT	Organotion	Antifouling	Male C57BL/6 mice	Activate PPAR*γ*, increase lipid accumulation and the expression of lipid metabolism	2021	Jie et al. [65]
	Dieldrin	Organochlorine	Insecticide	Male C57BL/6 mice	No affect the genes regulated by PPAR*γ* in hepatocarcinogenesis	2020	Wang et al. [97]
	Paraquat	Dipiridile	Herbicide	Male Wistar rats	Activation of PPAR*γ* with pioglitazone, decreases the concentrations of MDA (a lipid peroxidation marker)	2020	Amin et al. [107]
	Monocrotophos	Organophosphate	Insecticide	Male CFT-Wistar rats	Increase lipid content in the liver, PPAR*γ*, ACC, and FAS	2020	Nagaraju et al. [85]
	TPT	Organotion	Antifouling	*Xenopus tropicalis* embryos	TPT exposure reversed some impacts induced by PPAR*γ* overexpression	2018	Zhu et al. [110]
	TBT	Organotion	Antifouling	Female Wistar rats	Abnormal ovarian adipogenesis with increased cholesterol levels, lipid accumulation, PPAR*γ*, C/EBP-*β*, and Lipin-1	2018	de Araújo et al. [77]
	TBT	Organotion	Antifouling	Male C57bL/6J mice	Increase mRNA expression of the PPAR*γ* target genes Fabp4, Plin1	2017	Baker et al. [76]
	Mancozeb	Dithiocarbamate	Fungicide	Swiss albino mice	Affect PPAR*γ* and increased the cholesterol and TG	2014	Bhaskar and Mohanty [78]
	Imidacloprid	Neonicotinoid	Insecticide		No affinity to PPAR*γ*		
	Paraquat	Dipiridile	Herbicide	Male Wistar rats	Atorvastatin reduces the inflammation produced by pesticide, via PPAR*γ*	2014	Malekinejad et al. [131]
	Pronamide	Benzamide	Herbicide	Male CD-1 mice	The MoA of hepatocarcinogenesis although to PPAR*γ* and CAR	2014	LeBaron et al. [100]
	Nitrofen	Diphenyl ether	Herbicide	Pregnant rats and their fetus	Down-regulated PPAR*γ* and altered late gestation possibly due to impair lung development and maturation	2012	Gosemann et al. [132]
	Paraquat	Dipiridile	Herbicide	PPAR*γ* heterozygous mice (PPAR^clox/lox^/aP2-Cre)	Reduce expression of PPAR*γ*, improve insulin sensitivity, and increased resistance to paraquat-induce oxidative stress	2008	Luo et al. [105]
	TBT	Organotion	Antifouling	Pregnant C57BL/6J mice and their pups	Increase the number of adipocytes and lipid accumulation through RXR and PPAR*γ*	2006	Grün et al. [130]
PPAR*α* PPAR*γ*	Imidacloprid	Neonicotinoids	Insecticide	Zebrafish (*Danio rerio*)	Inhibit the growth of zebrafish and alters the levels of glycolipid metabolism and oxidative stress; reduce the expression of PPAR*α* and PPAR*γ*	2021	Luo et al. [96]
	Endosulfan sulfate	Organochlorine	Insecticide	Pregnant CD-1 mice and their male pups	In high and low-fat diet, PPAR*α* and its target gene Cpt1a are increased, but not modify PPAR*γ*	2021	Yan et al. [87]
	Chlorpyrifos	Organophosphate	Insecticide	Male zebrafish (*Danio rerio*)	Decrease PPAR*α* and PPAR*γ*, due to lipid metabolism disorders that are associated with gut oxidative stress and microbiota dysbiosis	2019	Wang et al. [86]
	Atrazine	Triazine	Herbicide	Male Kunming mice	Induce nephrotoxicity via modulating CYP450, PPAR*α*, PPAR*γ*, AhR, CAR, and PXR	2018	Xia et al. [108]
	Lambda cyhalothrin	Pyrethroid	Insecticide	Male albino rats	Up-regulate mRNA expression levels of PPAR*α*, PPAR*γ*, TNF-*α* FAS, and SREBP-1C	2016	Moustafa and Hussein [81]
	Triphenyltin	Organotion	Antifouling	Wood frog (*Lithobates sylvaticus)*	In chronic exposure, increase the expression of PPAR*α*, PPAR*γ*, FAS, and LPL	2013	Higley et al. [75]
PPAR*α* PPAR*γ* PPAR*β*/*δ*	Glyphosate	Organophosphate	Herbicide	Tilapia (*Oreochromis niloticus*)	Increase lipid content, alter redox status in liver, the genes involved in ion transport, lipid metabolism, and PPAR signaling pathway	2022	Jia et al. [74]
	Allethrin	Pyrethroids	Insecticide	Male Sprague Dawley rats	No activation of nuclear receptor in liver	2019	Fujino et al. [30]
	Bioresmethrin						
	Cis-permetryn						
	Cypermethrin						
	Deltamethrin						
	Fenvalerate						
	Trans-permetryn						
	Phenothrin						
	Difenoconazole	Triazole	Fungicide	Marine medaka (*Oryzias melastigma)*	Increase the expression of receptor PPAR*α*, PPAR*β*/*δ*, PPAR*γ*, and increase lipid levels in muscle but not in liver	2016	Dong et al. [73]
	Paclobutrazol	Triazole	Fungicide	Male rockfish (*Sebasticus marmoratus*)	Increase total lipid, TG, TC, free fatty acid and up-regulate PPAR*α*, PPAR*β*/*δ*, PPAR*γ*, AR, FAS, FABP4, ACC	2013	Sun et al. [72]
	Atrazine	Triazine	Herbicide	CFI mice	No interact with the receptors *α*, *β*/*δ*, or *γ*	2003	Devos et al. [104]
	Diclofop	Ariloxiphenoxypropionate	Herbicide	Male Wistar rats (Pzh:WIS)	Increase the number of peroxisome and are a rodent PP	2001	Palut et al. [101]
	Oxadiazon	Oxadiazol	Herbicide	Male SD rats	Peroxisome proliferation only occurred in rats and mice maybe to PPARs activation	1996	Richert et al. [83]
				Male CD1 mice			
				Male beagle dogs			

Abbreviations: 2,4-D, 2,4-Dichlorophenoxyacetic acid; ACC, acetyl Co-A carboxylase; ACO, acyl-CoA oxidase; AhR, aryl hydrocarbon receptor; AR, androgen receptor; AOX, alternative oxidase; C/EBP-*β*, CCAAT enhancer binding protein beta; CAR, constitutive androstane receptor; CPT-1, carnitine palmitoyltransferase I; FABP4, fatty acid-binding protein 4; FAS, fatty acid synthase; KO, knock out; LPL, lipoprotein lipase; MDA, malondialdehyde; PP, peroxisome proliferator; PPAR*α*, peroxisome proliferator-activated receptor alpha; PPAR*β*/*δ*, peroxisome proliferator-activated receptor beta or delta; PPAR*γ*, peroxisome proliferator-activated receptor gamma; PXR, pregnane X receptor; SD, Sprague Dawley; SREBP-1C, sterol regulatory element-binding protein 1; TBT, tributyltin; TC, total cholesterol; TG, triglycerides; TNF-*α*, tumor necrosis factor alpha; TPT, Triphenyltin.

## Data Availability

The review data supporting this Systematic Review are from previously reported studies and datasets, which have been cited. The processed data are available at the U.S. National Institutes of Health's National Library of Medicine (NIH/NLM).

## Abbreviations

2,4-D: 2,4-Dichlorophenoxyacetic acid
ACC: Acetyl-CoA carboxylase
ACO: Acyl-CoA oxidase
AhR: Aryl hydrocarbon receptor
AMPK*α*: AMP-activated protein kinase alpha
AOX: Alternative oxidase
AR: Androgen receptor
Arg: Arginine
ATP: Adenosine triphosphate
BM-MSC: Bone marrow-derived multipotent stromal cells
C/-EBP*α*: CCAAT enhancer-binding protein alpha
C/-EBP*β*: CCAAT enhancer-binding protein beta
C/-EBP*δ*: CCAAT enhancer-binding protein delta
C/EBPAs: CCAAT-enhancer-binding proteins
cAMP: Cyclic adenosine monophosphate
CAR: Constitutive androstane receptor
TCA: Tricarboxylic acid cycle
COS-1 cells: Fibroblast-like derived from monkey kidney
CPO: Chlorpyrifos-oxon
CPT-1: Carnitine palmitoyltransferase I
CREB: cAMP response element-binding protein
CS: Citrate synthase
CV-1 cells: Fibroblast cells from the kidney of an African green monkey
Cys: Cysteine
DDD: Dichloro diphenyldichloroethane
DDE: Dichloro diphenyldichloroethylene
DDT: Dichloro diphenyltrichloroethane
DEGs: Differentially expressed genes
DHHP: Diphenyl phosphate
DNA: Deoxyribonucleic acid
eCB: Metabolites of endocannabinoids
ecCTB cells: Extravillous trophoblast cell
EHDPP: 2-ethylhexyl diphenyl phosphate
EPN: Ethyl p-nitrophenyl phenylphosphorothioate
ER: Endoplasmic reticulum
FAAH: Fatty acid amide hydrolase
FADS2: Fatty acid desaturase 2
FAPB4: Fatty acid binding protein 4
FAS/FASN: Fatty acid synthase
FH: Fumarase
G3PD: Glycerol-3-phosphate
G6FDH: Glucose-6-phosphate dehydrogenase
GK: Glucokinase
GLUT-4: Glucose transporter type 4
GLUT-2: Glucose transporter type 2
GR: Glucocorticoid receptor
HCH: Hexachlorocyclohexane
HepaRG cells: Hepatic cells from human cholangiocarcinoma
His: Histidine
HK1: Hexokinase 1
IDH2: Isocitrate dehydrogenase
ILSI/PCS: The International Life Sciences Institute/International Program on Chemical Safety
KO: Knock out
LCFAs: Large-chain fatty acids
LDHB: Lactate dehydrogenase
Leu: Leucine
LpL: Lipoprotein lipase
LXR: Liver X receptor
Lys: Lysine
MCP1/CCL2: Monocyte chemotherapy protein 1
MCPA: 4-Chloro-o-toloxyacetic acid
MDA: Malondialdehyde
MEFs: Mouse embryo fibroblasts
MEHP: Mono-2(-ethylhexyl)-tetrabromophthalate
Met: Methionine
MoA: Mechanism of action
MSC: Mesenchymal stem cell
MUFAs: Monounsaturated fatty acids
NR: Nuclear receptors
OGDH: Oxoglutarate dehydrogenase
OP9 cells: Cell line derived from mouse bone marrow stromal cells
OPFRs: Organophosphate flame retardants
PDHA1: Pyruvate dehydrogenase alpha 1
PFKFB3: 6-Phosphofructo-2-kinase/fructose-2,6-biphosphatase 3
Phe: Phenylalanine
PK: Pyruvate kinase
PKC: Protein kinase C
PP: Peroxisome proliferators
PPAR*α*: Peroxisome proliferator-activated receptor alpha
PPAR*β*/*δ*: Peroxisome proliferator-activated receptor beta/delta
PPAR*γ*: Peroxisome proliferator-activated receptor gamma
PPARs: Peroxisome proliferator-activated receptors
PPRE: Peroxisome proliferator response elements
Pro: Proline
PUFAs: Polyunsaturated fatty acids
PXR: Pregnane X receptor
QpE: Quizalofop-p-ethyl
QSAR: Quantitative structure-activity relationship
RNA: Ribonucleic acid
ROS: Reactive oxygen species
RXR: Retinoid X receptor
SCD1: Stearoyl-CoA desaturase-1
SD: Sprague Dawley
SH-SY5Y cells: Thrice-subcloned cell line derived from the SK-N-SH neuroblastoma cells
SOD: Superoxide dismutase
SREBP-1c: Sterol regulatory element-binding protein 1
TAC: Tricarboxylic acid cycle
TBPH: Bis(2-ethylhexyl)-2,3,4,5-tetrabromophthalate
TBT: Tributyltin
TC: Total cholesterol
TCP: 3,5,6-Trichloropyridinol
TFs: Transcription factors
TG: Triglycerides
TNF-*α*: Tumor necrosis factor-alpha
TPHP: Triphenyl phosphate
TPT: Triphenyltin
WHO: World Health Organization
*β*-BHC: Benzene hexachloride beta
*γ*-BHC: Benzene hexachloride gamma
*δ*-BHC: Benzene hexachloride delta.
